# Perioperative Care and the Importance of Continuous Quality Improvement—A Controlled Intervention Study in Three Tanzanian Hospitals

**DOI:** 10.1371/journal.pone.0136156

**Published:** 2015-09-01

**Authors:** Goetz Bosse, Wiltrud Abels, Ferdinand Mtatifikolo, Baltazar Ngoli, Bruno Neuner, Klaus–Dieter Wernecke, Claudia Spies

**Affiliations:** 1 Department of Anaesthesiology and Intensive Care Medicine, Campus Charité Mitte and Campus Virchow-Klinikum, Charité—University Medicine Berlin, Berlin, Germany; 2 Bombo Regional Hospital, Tanga City, Tanga Region, Tanzania; 3 Component Leader Quality Improvement, Tanzanian-German Program To Support Health (TGPSH), giz Deutsche Gesellschaft für Internationale Zusammenarbeit GmbH, Dar-es-Salaam, Tanzania; 4 Charité—University Medicine Berlin, Germany and Sostana GmbH, Berlin, Germany; Azienda Ospedaliero-Universitaria Careggi, ITALY

## Abstract

**Introduction:**

Surgical services are increasingly seen to reduce death and disability in Sub-Saharan Africa, where hospital-based mortality remains alarmingly high. This study explores two implementation approaches to improve the quality of perioperative care in a Tanzanian hospital. Effects were compared to a control group of two other hospitals in the region without intervention.

**Methods:**

All hospitals conducted quality assessments with a Hospital Performance Assessment Tool. Changes in immediate outcome indicators after one and two years were compared to final outcome indicators such as Anaesthetic Complication Rate and Surgical Case Fatality Rate.

**Results:**

Immediate outcome indicators for Preoperative Care in the intervention hospital improved (52.5% in 2009; 84.2% in 2011, p<0.001). Postoperative Inpatient Care initially improved to then decline again (63.3% in 2009; 70% in 2010; 58.6% in 2011). In the control group, preoperative care declined from 50.8% (2009) to 32.8% (2011, p <0.001), while postoperative care did not significantly change. Anaesthetic Complication Rate in the intervention hospital declined (1.89% before intervention; 0.96% after intervention, p = 0.006). Surgical Case Fatality Rate in the intervention hospital declined from 5.67% before intervention to 2.93% after intervention (p<0.0010). Surgical Case Fatality Rate in the control group was 4% before intervention and 3.8% after intervention (p = 0.411). Anaesthetic Complication Rate in the control group was not available.

**Discussion:**

Immediate outcome indicators initially improved, while at the same time final outcome declined (Surgical Case Fatality, Anaesthetic Complication Rate). Compared to the control group, final outcome improved more in the intervention hospital, although the effect was not significant over the whole study period. Documentation of final outcome indicators seemed inconsistent. Immediate outcome indicators seem more helpful to steer the Continuous Quality Improvement program.

**Conclusion:**

Specific interventions as part of Continuous Quality Improvement might lead to sustainable improvement of the quality of care, if embedded in a multi-faceted approach.

## Introduction

Surgery is an integral part of global health care with an estimated 234 million major surgical procedures performed annually. In 2004, countries spending less than 100 US$ per head for health care accounted for a third of the global population. However, only 3.4% of all major surgery was carried out in these countries [1]. A similar disproportion had already been found 14 years earlier [2]. Today, essential surgical and anaesthetic services are increasingly acknowledged as possible key factors in reducing death and disability for developing countries, whilst remaining cost-effective [3–7].

In developing countries, mortality rates for patients undergoing major surgery have been suggested to range between 2.6% and 10%, depending upon hospital setting, type of anaesthesia and surgical procedure [1,8,9]. According to a review in 2012, perioperative mortality within 48 hours of surgery in developing countries was much lower than this figure [10]. However, the authors have been criticized for including too few studies from actual developing countries, thus underestimating mortality rates drastically [11]. Even in Europe, Pearse et al. found that average in-hospital mortality after non-cardiac surgery was as high as 4% with a broad confidence interval [12].

Searching for the reasons for high mortality rates, several authors have estimated that avoidable mistakes accounted for up to half of adverse events in surgical care in developing countries [13–15]. The World Health Organization stated that rates of avoidable deaths associated with surgery and anaesthesia in Sub-Saharan Africa depended upon the setting and could be 100–1000 times higher than in industrialized countries [16].

Avoidable mistakes can occur not only within, but also before and after the actual operation. In their 2012 review on perioperative mortality in developing countries, Bainbridge et al. defined the perioperative period as 48 hours after surgery [10]. Commonly, the perioperative period consist of pre-, intra-, and postoperative patient care. Preoperative care includes the preparation for surgery, including history taking and necessary diagnostics, documentation of risk factors, measures for limiting anxiety and adequate fasting orders. Intraoperative care includes the induction and maintenance of anaesthesia, monitoring of the patient and not at least the surgical procedure itself. Postoperative care begins with the admission to the recovery room, where vital signs are monitored closely, and does not end before discharge.

A study from the Netherlands found that the majority of critical incidents occurred pre- and postoperatively, not intraoperatively. Thus, it was emphasized that patient safety in the operating room alone, e.g. with the WHO Surgical Safety checklist, might be insufficient, when quality of care in the pre- and postoperative phases are neglected [17]. Traditionally, surgery has focused on the operation itself. However, only if we consider all three phases of perioperative care as equally important, we might be able to improve patient outcome. This accounts for both developed as well as developing countries.

The health care system in Tanzania is structured in levels, with health care facilities providing different levels of specification and emergency services. According to the Ministry of Health and Social Welfare in Tanzania, the lowest level is the village health service, followed by dispensary services, health centre services, district hospitals, regional hospitals and referral/consultant hospitals [18]. All hospitals in this study are district and regional hospitals, with emergency and surgical services. A study from 2011 reports severe structural shortcomings and lack of personnel in facilities providing emergency services and surgical services in Tanzania [19].

In 2005, the authors implemented the Hospital Performance Assessment Tool (HPAT) in all governmental hospitals in Tanga Region, Tanzania, to review the quality of health care services and identify areas in need of improvement [20]. As introduced in earlier studies, the results of the HPAT assessments can be considered as immediate outcome indicators as they assess the adherence to a target standard and give a result for an individual process immediately after it has ended [21]. Immediate outcome indicators augment final outcome indicators like e.g. mortality or case fatality, such as the Surgical Case Fatality Rate (SCFR). Next to structure and process, both kinds of outcome indicators are part of the HPAT assessments.

In 2009, a continuous quality improvement (CQI) approach was implemented in the intervention hospital by the Tanzanian partners. CQI is based on the idea of the Deming cycle [22] and has similarities with the Plan-Do-Study-Act (PDSA) cycle as outlined by the National Health Service (NHS) [23]. CQI aims to gradually improve care quality through a circle of: quality assurance, analysis of results, designing target-specific interventions, implementing change and re-evaluating effects through re-assessment (quality assurance) again. The approach has been suggested to potentially be valuable in developing countries, where challenges were multifaceted and often difficult to identify [24]. In our CQI approach, a detailed list of interventions (action plan) was implemented as a result of every assessment in the regional hospital over the period 2009–2011. A quality team (QT) concerned with monitoring the implementation of quality interventions was appointed from the hospital’s health workers.

In 2009, within the feedback meeting in the intervention hospital, the newly founded quality team identified several aspects of surgical care in their institution that were in need of improvement:

- In preoperative care, a preoperative visit was virtually non-existent, leading to a lack of information about any past medical history, bleeding disorders, medication or allergies. It was supposed that this was likely to entail complications during anaesthesia and contributed to the high anaesthetic complication rate in the hospital.
- In postoperative care, it was found that wound dressings were not changed at appropriate intervals, supposedly increasing infectious complications and eventually mortality; vital signs and urine input and output was not measured sufficiently, potentially leading to the non-detection of blood loss after major surgery or renal failure, which was considered to be associated with a high Surgical Case Fatality Rate (SCFR).

Concerning these challenges in pre- and postoperative care, the quality team decided on two specific interventions. In the field of preoperative care, a compulsory standardized checklist for the preoperative anaesthetic visit was introduced. For postoperative care, pre-existing weekly educational sessions were amended by topics from the clinical process of postoperative care, such as changing wound dressings, input/output documentation, and management of nasogastric tubes.

### Aim of the study

The aim of this study was to assess the effects of different quality improvement interventions and their respective implementation strategies in pre- and postoperative care between 2009 and 2011 in the intervention hospital. They were compared to a control group of two hospitals in the region with quality assessments but without interventions. Immediate and final outcome indicators (Surgical Case fatality Rate and Anaesthetic Complication rate) were used to evaluate quality changes.

## Methods

This study builds upon two previous studies by the same authors [20,21]. While the first two studies introduced the HPAT instrument and evaluated feasibility, this study goes beyond that by specifically investigating targeted interventions that had been identified through the annual hospital performance assessment and were specifically designed to improve quality of services in the intervention hospital.

### Ethical approval

In this prospective, controlled study data authorization and ethical committee approval was obtained from the National Institute of Medical Research in Tanzania as an amendment to ethical clearance reference number NIMR/HQ/P.12/Vol.VIII/27. For the routine collection of quantitative assessment data in the framework of the annual quality assurance it was decided by the ethic committee that it was not necessary to further obtain patients’ written consent. The assessments were part of the internal quality assessment by the hospital management and no patient had to undergo additional and/or invasive procedures.

### Study setting

Tanga Region is in Northeastern Tanzania. In the 2012 census, the population of the region was counted to be 2,045,205 people. There are six governmental hospitals in the region providing emergency and surgical services as district and regional hospitals. The intervention hospital is the regional hospital, and as such the reference institution for the whole region. According to the Ministry of Health in Tanzania, the difference between district hospital and regional hospital is supposed to be a higher density of specialist services [18]. Indeed, the intervention hospital provides e.g. ophthalmological services and advanced lab services. However, during the study period, there was no specialist general surgeon present in the intervention hospital. Between 2009 and 2011, the most common surgical diagnoses for inpatients in the intervention hospital were hernia and varicocele, appendicitis, intestinal obstruction and septic wounds. Almost 50% of the operations were emergency procedures. At baseline in 2009, all hospitals in this study sometimes had more patients than beds on the surgical wards. Antibiotic suspension solutions were shared among patients, if available. Drug sideboards were rarely locked, but anyways almost always empty, and patients were required to buy their medication themselves from the pharmacy on the hospital grounds. Disinfection was not always available, neither were gloves. Side rooms and toilets were often dirty, bedpans or–buckets had to be emptied into shared toilets and examination tables were not cleaned after use. Blood pressure machines were not always functioning well, if available at all. There was no intensive care unit in none of the hospitals. In the files of surgical patients, allergies, medication and past medical history was only irregularly documented in all of the hospitals. Postoperatively, monitoring of vital signs like blood pressure and fever as well as urine input/output was almost never documented as often as the orders required.

### Intervention hospital and control group

There are six governmental hospitals in Tanga Region, five district hospitals and one district/regional hospital. The district/regional hospital is the reference institution for all other hospitals in the region. As the regional hospital, it is supposed to provide more specialized care and a higher level of expertise. It is also bigger than the other hospitals. However, during the course of this study there was no specialized general or orthopaedic surgeon working at the regional hospital. Thus, it mainly served as the district hospital for the largest city in the region with roughly the quarter of a million inhabitants. The main difference between the intervention hospital and the district hospitals was the administrative function, which primarily did not affect clinical procedures. The Hospital Performance Assessment Tool (HPAT) used in this study can be used to assess clinical procedures in district and regional hospitals. In this study, the regional hospital will be referred to as the intervention hospital, because it was the only hospital with a full continuous quality improvement (CQI) approach. In the other five hospitals in Tanga Region, there were only annual assessments of care quality and feedback meetings, but no interventions to improve areas with low performance or other quality improvements efforts. One of the district hospitals was not included into the control group because it was a former missionary hospital thus being different in structural quality and process organization. In another district hospital there had been an attempt to introduce an action plan in 2009 that had not been followed up. At baseline, only two of the remaining three hospitals were similar in terms of bed capacity and number of major surgical operations. Hence, the control group eventually consisted of these two district hospitals. They were pooled together in the control group of this study to allow comparison to the intervention hospital. The groups were comparable in number of beds and number of operations, number of medical personnel and number of surgical wards. However, a detailed list of individual hospital characteristics has been given in the results section and limitations are discussed later in the manuscript (Table 1). A similar comparison of a control group with the intervention hospital has been used in an earlier study [21].

**Table 1:** Individual hospital characteristics of the control group 2009 to 2011.

Hospital characteristics	2009	2010	2011
**Intervention hospital**			
Bed Capacity	392	392	392
Number of health workers	370	382	382
Medical Doctors / Assistant Medical Officers	27	33	33
Number of major surgical operations	1495	1558	1558
**Control group total**			
Bed Capacity	220	120	229
Number of health workers	159	197	286
Medical Doctors / Assistant Medical Officers	16	15**	31
Number of major surgical operations	1211	1574	1775
**Control group hospital 1**			
Bed Capacity	119	120	120
Number of health workers	36*	34*	120
Medical Doctors /Assistant Medical Officers	7	1**	12
Number of major surgical operations	594	975	1112
**Control group hospital 2**			
Bed Capacity	101	109	109
Number of health workers	123	163	166
Medical doctors / Assistant Medical Officers	9	14	19
Number of major surgical operations	617	599	663

Figures were taken from the annual hospital reports of the intervention hospital and the regional primary health care (PHC) reports (please see supplementary files: S1 Table, S2 Table, S3 Table, S4 Table, S5 Table, S6 Table, S7 Table)

*the number of health workers seems inconsistently documented (supplementary file: S1 Table, S2 Table)

** the number of medical doctors in the second control group hospital is missing in 2010, thus this number is likely to be underestimated (see supplementary file: S5 Table).

### Hospital Performance Assessment Tool (HPAT) and continuous quality improvement

All assessments in this study have been performed with a quality assessment tool, which has been published by the authors in earlier articles, the Hospital Performance Assessment Tool (HPAT) [20,21]. The HPAT is structured along Donabedian’s structure, process and outcome (SPO) model of quality [25]. It includes 1162 items organized in 55 key procedures for process quality and 11 procedures for structural quality. Key procedures are based on evidence-based recommendations and national as well as international guidelines. They were carefully adapted to the local setting before their first application in 2006 in Tanga region. This is in line with Bainbridge et al. who have called for evidence-based best practice in developing countries in order to reduce the total perioperative and anaesthesia-related mortality [10]. Thus, quality of services is assessed against a target standard. In the surgical part of the HPAT there are a total of 12 key procedures with 176 items [21]. For every key procedure there is a checklist that is used for on-site observation and/or patient file review. The result of every key procedure is an immediate outcome indicator that specifically reflects the key procedure it derives from. The immediate outcome indicator expresses to what extend the target standard expressed with the checklist is adhered to. E.g., one immediate outcome indicator in this study is if wound dressings are changed according to orders. If the indicator is fulfilled it will help to detect wound infections and initiate timely treatment. Thus, the interaction of all immediate outcome indicators, along with hospital-independent factors leads to the final outcome, e.g. the Surgical Case Fatality Rate (CFR). Final outcome indicators in this study are Anaesthetic Complication Rate (ACR) and Surgical Case Fatality Rate (SCFR). They were derived from the annual hospital reports of the intervention hospital, from the annual regional hospital reports, and from the documentation of the surgical departments in the control group hospitals. The distinction between immediate and final outcome indicators has been established and described in detail in an earlier publication [21].

All HPAT assessments were part of ongoing routine annual quality assessments that have been conducted in all hospitals in Tanga region since 2006. Part of the HPAT approach are feedback meetings after every assessment, where results were analysed, presented and discussed with the health workers from the respective facility. Assessments and feedback meetings have been conducted every year in all hospitals since 2006. In the 2009 feedback meeting in intervention hospital, the hospital management decided to use assessment results to not only identify areas with low performance but also improve them with specific quality improvement interventions. A quality team was appointed from all groups of health workers, as described in a previous study [21]. The team was to identify areas with low performance, design specific interventions, implement them and evaluate changes in the next HPAT assessment feedback meeting one year later. This approach is similar to the plan-do-study-act cycle [23], the Deming cycle [22,26] and constitutes a continuous quality improvement (CQI) approach. All the other hospitals in the study did not introduce such an approach, although they would have had the possibility.

### Specific interventions for pre- and postoperative care

In the field of surgery, two aspects of surgical care were found in need of improvement, namely pre- and postoperative care. As described in the introduction, a preoperative visit was virtually non-existent, leading to a lack of information about the patient’s past medical history, bleeding disorders, medication or allergies. It was supposed that this was likely to entail complications during anaesthesia and contributed to the high anaesthetic complication rate in the hospital. In postoperative care, it was found that wound dressings were not changed at appropriate intervals, supposedly increasing infectious complications and eventually mortality; vital signs and urine input and output was not measured sufficiently, potentially leading to the non-detection of blood loss after major surgery or renal failure, which was considered to be associated with a high Surgical Case Fatality Rate (SCFR). These aspects were reflected in two key procedure checklists in the HPAT. One was the checklist “preoperative care, the other was a combination of items from the existing checklists “postoperative care”, “ward round”, and “service quality”, which was termed “postoperative inpatient care” for this study. The latter included amongst others the changing of wound dressings at adequate intervals, the documentation of input and output, the application of pain medication, and the proper use of nasogastric tubes.

The interventions designed to meet these challenges were:

- For preoperative care: The introduction of a compulsory standardized checklist for the anaesthetic visit the day before surgery. The underlying implementation approach was multi-faceted and integrated several characteristics of a successful implementation [27,28]: provision of a) an evidence-based target standard for the measurement of clinical performance, b) an educational tool for on-the-job training, and c) a matrix for supervision and feedback.
- For postoperative care: The introduction of educational sessions on selected items in the performance of postoperative care, such as: application of pain medication, management of nasogastric tubes, wounds dressings, and documentation of intake and output in patient files. The implementation approach here was to amend a pre-existing continuing education program in the framework of the weekly department meetings.

The implementation of interventions was to be conducted immediately and changes were to be evaluated in the next HPAT assessment. The head surgeon and head nurse were accountable for the implementation within the department and provided supervision for the use of the checklist in preoperative care, as well as gave the presentation on postoperative care.

### Assessment method

**Fig 1:**
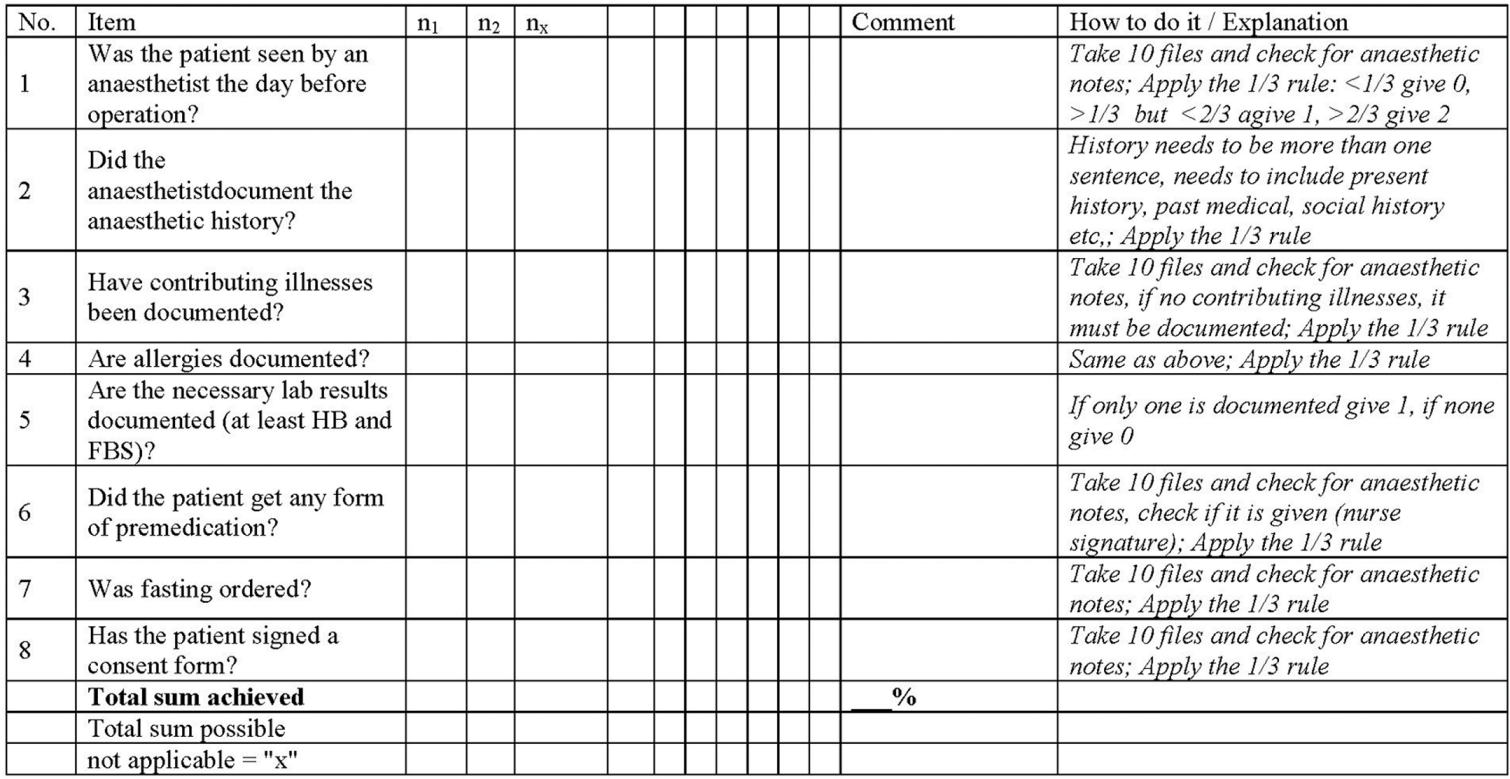
HPAT assessment checklist for Preoperative Care. Whenever possible, ten observations or file reviews should be conducted for key procedures in process quality. Single items were assessed with 0/2 = not available/performed = 0%, 1/2 = partly available/irregularly performed = 50% or 2/2 = available/performed = 100%.

**Fig 2:**
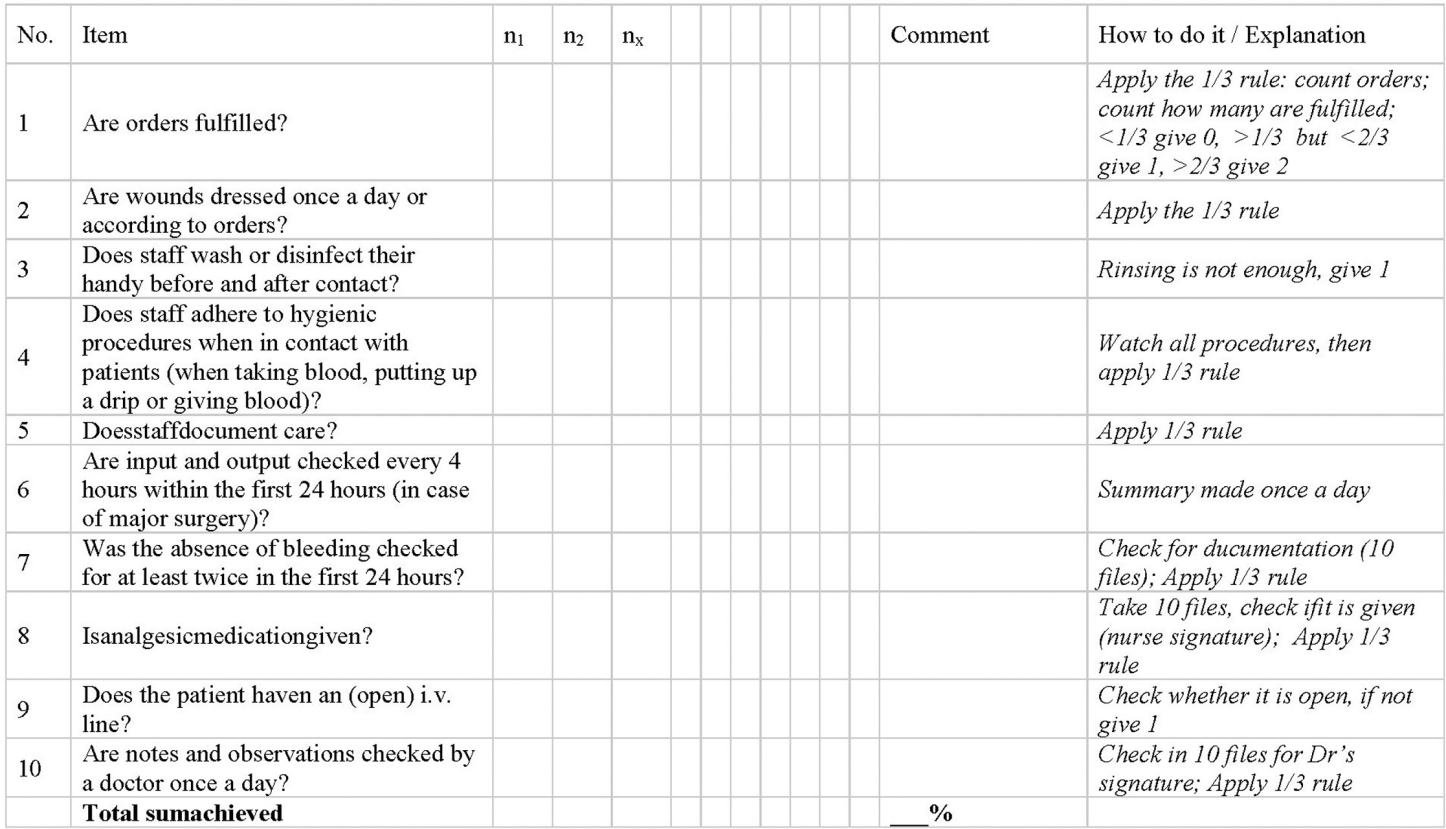
HPAT assessment checklist for Postoperative Inpatient Care. Whenever possible, ten observations or file reviews should be conducted for key procedures in process quality. Single items were assessed with 0/2 = not available/performed = 0%, 1/2 = partly available/irregularly performed = 50% or 2/2 = available/performed = 100%.

Both key procedures “preoperative care” and “postoperative inpatient care” consist of a checklist of items. Both procedures are given in Fig 1 and Fig 2.

Preoperative care had 8 items, postoperative inpatient care had 10 items. Checklist items were assessed on a Likert scale with either 0, 1, or 2: Zero (0) expressed 0% of the standard was adhered to and the item was not performed/not available. One (1) expressed 33.3%–66.6% of the standard was adhered to and the item was irregularly performed/not always available. Two (2) expressed 66.67%–100% of the standard was adhered to or fully performed/always available. E.g., if wound dressing were to be changed every day over the course of 7 days, than a “0” was given, when it was done less than twice; a “1” was given when it was done more often than twice but less than 5 times; a 2 was given when it was done 5 times and more often. The application of the rule for specific items was described in a “How to do it” section within the assessment checklists. This column explains explicitly how every item was to be assessed, and a cut off level was described for every individual item. Assessments were conducted in the same fashion in all hospitals in this study. Two observers assessed all items individually and compared their results later on in order to limit observer bias. In case of disagreement, assessments were repeated. They were supervised by the Regional Health Management Team. Assessments were conducted annually and took an average of three working days in all hospitals, respectively.

Immediate outcome indicators for the two key procedures under survey in this study are given as average percentage of all items. Amongst others, Nachtigall et al. have described that 70% of adherence to standards can improve patient outcome [24]. In our approach, it was agreed within the quality team that 80% was the benchmark for good quality, as described in a previous publication [21].

### Comparison of results

Differences in immediate outcome indicators from the baseline (2009) and within the intervention period 2010 to 2011 were compared over time as well as between intervention hospital and control group. Surgical Case Fatality Rates (SCFR) was calculated from the annual hospital reports of the intervention hospital. It was compared over time and between intervention hospital and control group. Anaesthetic Complication Rate was calculated from the annual report of the intervention hospital and compared over time.

### Statistical analysis

Quantitative results were expressed and compared as fractions and proportions [%]. Confidence intervals for changes in immediate outcome indicators were given. In addition, the significance of changes in immediate outcome indicators was tested with chi-square-tests. All tests were carried out in exact versions because of small and unbalanced sample sizes. A two-tailed p<0.05 was considered statistically significant. Analysis was done with IBM SPSS Statistics, Version 21, IBM Corporation and other(s) 1989, 2012. In order to relate immediate outcomes with final outcomes, data were stratified into years. Confidence intervals for the differences were given. Significance of changes in stratified final outcome indicators were tested with the Breslow and Day test for homogeneity of odds ratios in the strata as well as the Mantel-Haenszel test, as appropriate. Testing was done with StatXact, Version 6, CYTEL Software Corp., Cambridge, MA 02139, USA.

## Results

### Hospital characteristics

See Table 1.

### Structural quality

Immediate outcome indicators for structural quality in the surgical department and the operating theatre of the intervention hospital did not significantly improve. In the control group, structural quality declined over the study period from 67.1% (2009) to 50% (2011, p = 0.003). For overall structural quality in the intervention hospital and the control group see Tables 2 and 3.

**Table 2:** Structural quality in the intervention hospital 2009 to 2011.

Intervention hospital Structural quality	2009 (t_1_) [%]	2010 (t_2_) [%]	2011 (t_3_) [%]	p value t_1_—t_2_	p value t_2_ –t_3_	p value t_1_ –t_3_
Surgical department	44/76 [57.9]	148/228 [64.9]	156/224 [69.6]	0.38	0.546	0.11

Significance was tested 2010 (t2) against 2009 (t1), 2011 (t3) against 2010 (t2) and 2011 (t3) against 2009 (t1).

**Table 3:** Structural quality in the control group 2009 to 2011.

Control group Structural quality	2009 (t_1_) [%]	2010 (t_2_) [%]	2011 (t_3_) [%]	p value t_1_—t_2_	p value t_2_ –t_3_	p value t_1_ –t_3_
Surgical department	102/152 [67.1]	75/150 [50]	75/150 [50]	0.003*↓.	1	0.003*↓.

Significance was tested 2010 (t2) against 2009 (t1), 2011 (t3) against 2010 (t2) and 2011 (t3) against 2009 (t1). Significant decline was marked *↓.

### Process quality—Preoperative Care

Immediate outcome indicators for Preoperative Care in the intervention hospital improved significantly in the first year after intervention (52.5% (2009) to 78.4% (2010), p < 0.001 =) and remained stable after that (84.2% (2011), p = 0.15; please refer to Table 4). While in 2009, only 4 out of 10 patients received a documented preoperative visits by an anaesthetist, all assessed preoperative patients received a visit the day before operation in 2010 and 2011. Anaesthetic history was documented in 50% (10/20) in 2009, and in 100% (30/30) in 2011.

**Table 4:** Immediate outcome indicators for Preoperative Care in the intervention hospital 2009 to 2011.

Intervention hospital Preoperative Care	2009 t_1_ [%]	2010 t_2_ [%]	2011 t_3_ [%]	p value t_1—_t_2_	p value t_2 –_t_3_	p value t_1 –_t_3_
Was the patient seen by an anesthetist the day before operation?	8/20 [40]	22/22 [100]	30/30 [100]	< 0.001*↑	1	< 0.001*↑
Did she/he document the anesthetic history?	10/20 [50]	22/22 [100]	30/30 [100]	< 0.001*↑	1	< 0.001*↑
Have contributing illnesses been documented?	8/20 [40]	9/22 [40.9]	20/30 [66.7]	1	0.092	0.085
Are allergies documented?	10/20 [50]	0/22 [0]	17/30 [56.7]	< 0.001*↓	< 0.001*↑	0.774
Are the necessary lab results documented (at least HB and FBS)?	8/20 [40]	22/22 [100]	15/30 [50]	< 0.001*↑	< 0.001*↓	0.569
Did the patient get any form of premedication?	4/20 [20]	8/8 [100]	30/30 [100]	< 0.001*↑	1	< 0.001*↑
Was fasting ordered?	16/20 [80]	22/22 [100]	30/30 [100]	< 0.043*↑	1	< 0.001*↑
Did the patient sign a consent form?	20/20 [100]	22/22 [100]	30/30 [100]	1	1	1
overall score [%]	84/160 [52.5]	127/162 [78.4]	202/240 [84.2]	< 0.001*↑	0.149	< 0.001*↑
difference of scores				-0.2590	-0.0577	-0.3167
95% CI of differences				-0.3544 –-0.1560	-0.1383–0.0187	-0.4038 –-0.2249

Immediate outcome indicators from 2009, 2010, 2011. 2009 is the baseline assessment. Significance was tested 2010 (t2) against 2009 (t1), 2011 (t3) against 2010 (t2) and 2011 (t3) against 2009 (t1). Significant improvement is marked with *↑. Significant decline was marked *↓. Confidence interval is given for the differences of overall immediate outcome of the key procedure in 2009, 2010 and 2011.

In the control group, the quality of Preoperative Care did significantly decrease over the whole study period (see Table 5). Patients received anaesthetic visits the day before operation in less than 25% over the whole study period (7/32 in 2009, 0/28 in 2010 and 2011).

**Table 5:** Immediate outcome indicators for Preoperative Care in the Control Group 2009 to 2011.

Control group Preoperative Care	2009 t_1_ [%]	2010 t_2_ [%]	2011 t_3_ [%]	p value t_1—_t_2_	p value t_2 –_t_3_	p value t_1 –_t_3_
Was the patient seen by an anesthetist the day before operation?	7/32 [21.9]	0/28 [0]	0/28 [0]	0.012*↓	1	0.012*↓
Did she/he document the anesthetic history?	22/32 [68.8]	0/28 [0]	0/28 [0]	<0.001*↓	1	<0.001*↓
Have contributing illnesses been documented?	16/32 [50]	0/28 [0]	20/28 [71.4]	<0.001*↓	<0.001*↑	0.117
Are allergies documented?	9/32 [28.1]	0/28 [0]	0/28 [0]	0.002*↓	1	0.002*↓
Are the necessary lab results documented (at least HB and FBS)?	25/32 [78.1]	8/28 [28.6]	16/28 [57.1]	<0.001*↓	0.058	0.101
Did the patient get any form of premedication?	4/32 [12.5]	0/28 [0]	0/28 [0]	0.116	1	0.116
Was fasting ordered?	22/32 [68.8]	3/28 [10.7]	13/28 [46.4]	<0.001*↓	0.007*↓	0.116
Did the patient sign a consent form?	25/32 [78.1]	14/28 [50]	14/28 [50]	0.031*↓	1	0.031*↓
overall score [%]	130/256 [50.8]	25/192 [13.02]	63/192 [32.8]	<0.001*↓	<0.001*↑	<0.001*↓
difference of scores				0.3776	-0.1979	0.1797
95% CI of differences				0.2956–0.4505	-0.2781 –-0.1147	0.0875 –-0.2668

Immediate outcome indicators from 2009, 2010, 2011. 2009 is the baseline assessment. Significance was tested 2010 (t2) against 2009 (t1), 2011 (t3) against 2010 (t2) and 2011 (t3) against 2009 (t1). Significant improvement is marked with *↑. Significant decline was marked *↓. Confidence interval is given for the differences of overall immediate outcome of the key procedure in 2009, 2010 and 2011.

### Process quality—Postoperative Inpatient Care

Immediate outcome indicators for Postoperative Inpatient Care in the intervention hospital improved in the first year after the start of the intervention, although not significantly (63.3% (2009) to 70% (2010), p = 0.33; please refer to Table 6). In 2011, the overall level of performance quality declined again to a level comparable to the baseline (58.6% (2011), p = 0.49). E.g., input and output were checked in 50% (4/8) in 2009, 55% (11/20) in 2010 and 40.1% (9/22) in 2011. However, the changing of wound dressing improved from 50% (1/2) in 2009 to 83.3% (5/6) in 2010 to 100% (6/6) in 2011.

**Table 6:** Immediate outcome indicators for Postoperative Inpatient Care in the intervention hospital 2009 to 2011.

Intervention Hospital Postoperative Inpatient Care	2009 t1 [%]	2010 t2 [%]	2011 t3 [%]	p value t1—t2	p value t2 –t3	p value t1 –t3
Are orders fulfilled?	1/2 [50]	5/6 [83.3]	5/6 [83.3]	1	1	1
Are wounds dressed once a day?	1/2 [50]	5/6 [83.3]	6/6 [100]	1	1	1
Does staff wash or disinfect their hands before and after contact?	0/2 [0]	4/6 [66.7]	2/6 [33.3]	0.429	0.567	1
Does staff adhere to hygienic procedures when in contact with patients (when taking blood, putting up a drip or giving blood)?	1/2 [50]	6/6 [100]	4/6 [66.7]	0.25	0.455	1
Does staff document care?	1/2 [50]	4/6 [66.7]	3/6 [50]	1	1	1
Are input / output checked every 4 hours for the first 24 hours?	4/8 [50]	11/20 [55]	9/22 [40.1]	1	0.537	0.698
Is absence of bleeding checked at least twice?	7/20 [35]	10/32 [31.3]	3/22 [13.7]	1	0.199	0.152
Is analgesic medication given?	20/20 [100]	32/32 [100]	22/22 [100]	1	1	1
Does the patient have an (open) iv line?	8/12 [66.7]	20/24 [83.3]	12/22 [54.5]	0.397	0.054	0.717
Are notes and observation charts checked by Dr and documented once a day?	12/20 [60]	21/32 [65.6]	16/22 [72.7]	0.771	0.767	0.515
overall score [%]	57/90 [63.3]	119/170 [70.0]	82/140 [58.6]	0.329	0.042*↓	0.493
difference of scores				-0.0667	0.1143	0.0476
95% CI of differences				-0.1879–0.0507	0.0075–0.2189	-0.0818–0.1716

Immediate outcome indicators from 2009, 2010, 2011. 2009 is the baseline assessment. Significance was tested 2010 (t2) against 2009 (t1), 2011 (t3) against 2010 (t2) and 2011 (t3) against 2009 (t1). Significant improvement is marked with *↑. Significant decline was marked *↓. Confidence interval is given for the differences of overall immediate outcome of the key procedure in 2009, 2010 and 2011.

In the control group, the quality of Postoperative Inpatient Care declined in 2010, and improved again in 2011 up to a level similar to 2009 (for all immediate outcome indicators, see Table 7).

**Table 7:** Immediate outcome indicators for Postoperative Inpatient Care in the Control Group 2009 to 2011.

Control group Postoperative Inpatient Care	2009 t_1_ [%]	2010 t_2_ [%]	2011 t_3_ [%]	p value t_1—_t_2_	p value t_2 –_t_3_	p value t_1 –_t_3_
Are orders fulfilled?	3/4 [75]	2/4 [50.0]	2/4 [50.0]	1	1	1
Are wounds dressed once a day?	2/4 [50]	2/4 [50.0]	2/4 [50.0]	1	1	1
Does staff wash or disinfect their hands after contact?	3/4 [75]	0/4 [0]	0/4 [0]	0.143	1	1
Does staff adhere to hygienic procedures when in contact with patients (when taking blood, putting up a drip etc.)?	3/4 [75]	2/4 [50.0]	2/4 [50.0]	1	1	1
Does staff document care?	3/4 [75]	2/4 [50.0]	2/4 [50.0]	1	1	1
Is the input /output checked every 4 hours for the first 24 hours (in case of major operation)?	17/34 [50]	6/18 [33.3]	19/26 [73.1]	0.379	0.014*↑	0.11
Is absence of bleeding checked at least twice?	15/34 [44.1]	3/18 [16.7]	7/26 [26.9]	0.068	0.489	0.036*↓
Is anti-pain medication applied?	54/54 [100]	17/18 [94.4]	26/26 [100]	0.25	0.409	n.a.
Does the patient have an (open) iv line?	47/54 [74]	18/18 [100]	26/26 [100]	0.181	1	0.09
Are notes and observation charts checked by Dr and documented once a day?	35/40 [87]	3/18 [16.7]	13/26 [50]	0.172	<0.001*↓	<0.001*↓
overall immediate outcome [%]	123/176 [69.9]	55/110 [50]	99/150 [66]	0.001*↓	0.011	0.476
difference				0.1989	-0.1600	0.0389
95% CI of differences				0.0825–0.3102	-0.2961 –-0.0389	-0.0618 –-0.1398

Immediate outcome indicators from 2009, 2010, 2011. 2009 is the baseline assessment. Significance was tested 2010 (t2) against 2009 (t1), 2011 (t3) against 2010 (t2) and 2011 (t3) against 2009 (t1). Significant improvement is marked with *↑. Significant decline was marked *↓. Confidence interval is given for the differences of overall immediate outcome of the key procedure in 2009, 2010 and 2011.

### Outcome quality—Final outcome indicators

The Surgical Case Fatality Rate (SCFR) in the intervention hospital declined significantly after 2009 (5.67% (before intervention) to 2.93% (after intervention), p<0.001; please refer to Table 8), but not in the control group (4% (before intervention) to 3.8% (after intervention), p = 0.411; please refer to Table 8).

**Table 8:** Surgical Case Fatality Rate for the intervention hospital and the control group before and after intervention.

	before intervention [%]	after intervention [%]	p value before—after
SCFR intervention hospital	5.67% n = 3,035	2.93% n = 3,654	<0.001*↓
	172/3035	107/3654	
Difference [%]			2.74
CI of difference [%]			1.77–3.75
SCFR control group total	4.0%	3.8%	0.411
	n = 11040	n = 13664	
	447/11040	525/13664	
p-value	< 0.001	0.009	
Difference [%]	1.62	-0.91	
CI of difference [%]	0.76–2.57	-1.52 –-0.24	
Odds Ratio [95%-CI]	1.42 [1.2–1.7]	0.76 [0.6–0.9]	
SCFR control group hospital 1	3.89% n = 6.831	3.73% n = 8.797	
	266/6831	328/8797	
	< 0.001	0.029	
Odds Ratio [95%-CI]	1.48 [1.2–1.8]	0.78 [0.6–0.9]	
SCFR control group hospital 2	4.3% n = 4209	4.04% n = 4867	
	181/4209	197/4867	
	0.008	0.007	
Odds Ratio [95%-CI]	1.34 [1.1–1.7]	0.72 [0.6–0.9]	

Figures were taken from the annual hospital reports, the annual regional reports and individual hospitals’ documentations. SCFR = Surgical Case Fatality Rate; before intervention = 2009; after intervention = 2010 or 2011, resp., as there was not always available in both years

The rate of anaesthetic complications in the intervention hospital declined significantly after 2009 (1.89% (before intervention) to 0.96% (after intervention), p = 0.006; please refer to Table 9). The rate of anaesthetic complications in the control group was not available over the whole study period.

**Table 9:** Anaesthetic Complication Rate for the intervention hospital before and after intervention.

	before intervention	after intervention	
ACR intervention hospital	1.89% n = 2,431	0.96% n = 2,710	0.006*↓
	46/2431	26/2710	
difference [%]			0.93
CI of difference [%]			0.29–1.63
ACR control group	n.a.	n.a.	

ACR = Anaesthetic Complication Rate. Significance was tested a) before intervention (2009) against after intervention (2010, as there was no data available for 2011). Significant decline was marked with *↓.

Stratified analysis of SCFR with respect to the years 2009 and 2011 resulted in significantly different odds ratios of strata (Breslow and Day test, p<0.0001), i.e. SCFR was different in those years between hospital and control (odds ratio before intervention 1.42, odds ratio after intervention 0.76). Details for the SFCR before and after intervention are given in Table 8. SCFR differed significantly between hospital and control both before and after intervention (p ≤ 0.01 each), but the common odds ratio over the years of 1.05 was not significant (p = 0.52, Mantel-Haenszel test).

## Discussion

The main findings of this study are:

1. Changes in structural quality were independent from changes in process quality.
2. Through the CQI approach, immediate outcome indicators for preoperative care in the intervention hospital improved. Improvements were associated with a decrease in the Surgical Case Fatality Rate and the Anaesthetic Complication Rate.
3. The comparison of pre- and postoperative implementation strategies in the intervention hospital showed that in the first year both implementation approaches lead to improvement in immediate outcome indicators. After two years, it seems that the improvement was not sustainable in Postoperative Inpatient Care.
4. Initial improvements in performance quality were associated with both decreased Surgical Case Fatality Rate and Anaesthetic Complication Rate in the intervention hospital. However, final outcome data were partly not available and documentation of final outcome indicators appeared questionable. Thus, final outcome indicators alone seem not sensitive enough a measuring and/or steering tool for changes and improvements in pre- and postoperative care in this setting.

### Structural quality

In our study, structural quality seemed to be hardly associated with process quality. Process quality did not always decrease or increase along with structural quality, let alone to the same amount. As Peabody et al. pointed out in 2006 for the World Bank, structural improvements by themselves rarely improve the health of a population in developing countries [29]. We agree with Reerink et al. who said that it was not sensible to exclusively focus on improving structural quality in order to improve patient outcome; but rather to strengthen process quality, as they are different entities [30,31].

### CQI and improvement

Through embedding HPAT results into a continuous quality improvement strategy, preoperative service quality in the intervention hospital seemed to improve lastingly over the course of the study. Postoperative interventions did only initially improve and declined again. These findings do not prove that integrating interventions into a continuous quality improvement program is a prerequisite for sustainability. However, they suggest it, as CQI has been proposed to be especially useful for developing countries, where deep-rooted challenges might have to be improved in a continuous process rather than resolved “overnight” [24]. In this study, CQI interventions in the intervention hospital lead to better immediate outcome indicators, while at the same time the Surgical Case Fatality Rate and the Anaesthetic Complication Rate decreased as compared to the control group.

### Comparison of implementation approaches

Through the implementation approach of introducing a compulsory standardized checklist, the intervention in preoperative care appears to have had a significant positive effect on the immediate outcome indicators, which lasted over the whole study periods. It is well known that checklists increase standardization in work processes and avoid reliance on memory, thereby decreasing the chances of human error [32]. In its 1999 report “To err is human”, the Institute of Medicine recommended verification processes such as checklists in order to minimize the human factor in complex medical processes [33]. We agree to that. However, we suggest that there are more reasons why the use a checklist has a good potential for sustainable improvement. Checklists combine several aspects of a successful implementation approach. They represent the target standard from which they derive. They can be applied as an educational tool, are available at the point of care, serve as a matrix for on-the-job-training, and give immediate feedback on completion of a task. They make performance measurable and can positively motivate in case of improved performance. They can be used for supervision and control of the actual performance in daily practice. Other large-scale interventions are reported, where checklists lead to quality improvements. As a well-known example, after its worldwide introduction, the Surgical Safety Checklist by the World Health Organization has been recognised as a generally effective measure in order to increase patient safety in the operating room [14]. In 2013, a Quality Rounds Checklist could sustainably increase compliance with evidence-based standards and decrease complications in a trauma intensive care unit [34]. Through its multifaceted character they might be a cornerstone to improve patient safety sustainably.

In Postoperative Inpatient Care the implementation strategy was to amend the pre-existing continuing education program. Follow-up, on-the-job-training, supervision or feedback mechanisms were not included. After an initial increase in performance, improvements could not be sustained. We believe that the introduction of educational sessions alone can only be the starting point or an accompanying feature of an intervention, but seems to lack important aspects to maintain improved performance. Several authors tried to describe those additional aspects. In a qualitative study on the implementation of a perinatal audit in South Africa, several factors were identified to play a central role for implementation and sustainability. Amongst those factors were supervisory activities and feedback meetings [27]. A study on the implementation of the guidelines for Integrated Management of Childhood Illnesses (IMCI) found that performance feedback was a promising technique for improving adherence to the guidelines and the quality of care [28]. In Kenyan district hospitals, Ayieko et al found that a “full intervention” in paediatric care with local facilitation, supervision and face-to-face feedback was associated with greater quality improvement after 18 months than a merely “partial” intervention where those aspects were absent [35].

In their 2012 review, Hulscher et al tested 23 studies from all over the world for determinants of sustainable quality improvement interventions. There was no significant factor that for sure guaranteed success and sustainable improvement. However, the chance of long-term success was higher, whenever data collection on specific areas in need of improvement continued [36]. We agree with this view. Thus, in our program, implementation approaches for interventions are multifaceted and interventions themselves are assessed and re-assessed annually.

Umar et al. found that it might be effective to adopt a rather slow “little-steps” approach involving health workers to develop a quality culture and a management scheme with targeted interventions from the scratch [37]. In Benin, trained workers performed better when they were supervised after they received training on Integrated Management of Childhood Illnesses (IMCI) guidelines [38]. Continuing education after training on IMCI guidelines improved adherence in Morocco [39]. Thus, although in our study the introduction of a checklist has been proven to be lastingly effective, it has to be pointed out that it alone might not automatically lead to sustainably improved clinical processes and hence outcome. Aveling et al. expressed similar criticism towards the surgical safety checklist [40]. It seems crucially important to not only introduce but to continuously support and supervise the use of a checklist in order to have a sustainable effect.

In essence, a multi-faceted approach and continuation seem valuable. We suggest this to be the true difference between introduction and implementation. The best intervention seems to be doomed to fail if it is not hold through, and it also seems of utmost importance to follow-up and re-evaluate existing interventions rather than break up and plan new ones.

### Availability of final outcome indicators

In this study, the documentation of final outcome indicators was not always found to be trustworthy. Final outcome indicators were not always available, and documentation in the hospital reports seemed oftentimes inconsistent. This has also been described by other authors. As early as 1990, Nordberg et al. stated that it was difficult to assess the outcome of surgical care in East Africa because of problems in recording and reporting [2]. In 2009, Weiser et al. still found that the lack of standardised and well documented outcome measures for surgical care in developing countries seemed to be a global challenge [41].

Outcome-based monitoring is still the gold standard worldwide and reflects the success of any quality improvement program. If the quality of final outcome indicators is not assured, it seems difficult to use them for monitoring, evaluating and steering health care improvement programs. Thus, documentation and exactness in this field is mandatory. According to Donabedian, final patient outcome indicators are the desired ends of health care and cannot be neglected [21,42]. Amongst others, the WHO-based Health Metrics Network has acknowledged the challenge of completing and standardizing the collection of useful outcome measures. Until this collection is complete and documentation is reliable, it might make sense to rely on immediate outcome indicators as they are faster to generate and reflect immediately the situation, which in return helps to steer the program.

### Limitations

There are some severe limitations to this study.

First, the hospitals in this study are not all of the same size. Technically, the intervention hospital is a regional reference hospital. In theory, a regional hospital should provide a greater level of expertise and a higher level of clinical and nursing care capability. According to the definition of tertiary care, there are supposed to be specialist services available at the regional hospital. This requirement was met in our intervention hospital in most of the departments. To name a few examples, the regional hospital provides specialist services such as dental surgery, has an ophthalmological outpatient department, and a specialist surgeon for gynaecology and obstetrics services. However, during our study period, there was no specialist general or orthopaedic surgeon working at the regional hospital. Thus the surgical department in the regional hospital was comparable to the surgical departments in the control group.

Still, the regional hospital served the biggest city in the region and probably has more severe cases than the control group hospitals. Beyond doubt comparisons in the quality of health services are limited if they are not derived from similar patient groups and usually, scoring systems are needed for defining individual risk levels. These depend e.g. upon age, severity of disease, co-morbidities and past medical history [43]. In this study, data was obtained from routine quality assessments and thus specific case mix adjustment has not been applied. It has also been said that when process measures are assessed for quality improvement, the problem of case mix largely ends where appropriate processes of care for specific patient groups have been defined [43]. This is the case in the structure of the HPAT, where high risk patients depend on different standards than non-risk patients. Different risk groups might require different standards; however, all patients require that their standard is adhered to. In this way, basic stratification took place. This has been described in earlier studies [21].

Secondly, it is likely that the Hawthorne effect played no small role in our approach, as it must be assumed to do in most settings where observations take place. However, we think that the Hawthorne effect might have weighed less over the years, as assessments have become more and more part of the clinical routine since they had started in 2006. Also, assessments are conducted as peer assessments, which might have further reduced the Hawthorne effect.

Self-assessments bear a risk that individuals might want to make “their” hospital’s services appear better than they are. Thus, we used a system of two observers making independent observations and agreeing on one assessment result after comparing their individual ones. They were also supervised by the Regional Health Management Team, and we counterchecked the observations with file reviews.

Only one hospital in this study introduced an intervention to improve the quality of services. However, the quality assurance approach was the same for all district hospitals in Tanga Region. All the hospitals have used the Hospital Performance Assessment Tool (HPAT) and held feedback meetings to reflect upon the results since 2006. In 2009, the hospital management of the intervention hospital decided to go further and designed targeted interventions to very specifically improve areas where quality of services was low. They were not advised to do so and also all other hospitals would have had the freedom to do this or introduce other quality improvement activities. In our view, this self-directed course together with the self-assessment approach in the HPAT could be a crucial prerequisite of successful and hopefully sustainable quality improvement.

## Conclusion

Both interventions showed initial improvements in the quality of care in the intervention hospital compared to the control group. These improvements in performance quality seemed independent from changes in structural quality. However, the introduction of a standardized checklist on preoperative care was followed by more lasting improvements than including specific activities in a pre-existing framework of educational sessions in the field of postoperative care. The checklist reflected the target standard, was always available at the point of care, and could be used for a matrix for further improvement activities. All these aspects together may have led to better results for a longer time than one single implementation aspect, such as education, for postoperative care. Further studies will be needed to test these categories.

Along with improvements in pre- and postoperative care, Anaesthetic Complication Rate and Surgical Case Fatality Rate in the intervention hospital did decline over the study period. These findings are limited by the fact that final outcome indicators oftentimes seemed unreliable and not consistently documented. Thus, some doubts remain that results could be incidental. Well documented final patient outcome indicators in hospital-dependent quality of care are still urgently needed because only good documentation can truthfully reflect changes in patient long-term outcome. They also may reflect the success of a quality improvement program.

## Supporting Information

S1 Table From control group hospital 1 regional report 2009. (DOCX)

S2 Table From control group hospital 1 regional report 2010. (DOCX)

S3 Table From control group hospital 1 regional report 2011. (DOCX)

S4 Table From control group hospital 2 regional report 2009. (DOCX)

S5 Table From control group hospital 2 regional report 2010. (DOCX)

S6 Table From control group hospital 2 regional report 2011. (DOCX)

S7 Table From intervention hospital reports 2009–2011. (PDF)
