# Correction: Perioperative Care and the Importance of Continuous Quality Improvement—A Controlled Intervention Study in Three Tanzanian Hospitals

**DOI:** 10.1371/journal.pone.0156206

**Published:** 2016-05-19

**Authors:** Goetz Bosse, Wiltrud Abels, Ferdinand Mtatifikolo, Baltazar Ngoli, Bruno Neuner, Klaus–Dieter Wernecke, Claudia Spies

There are errors in Tables 5 and 7. During review, the size of the control group was reduced from three to two hospitals. Tables 5 and 7 were not completely updated to reflect this change. Please see the corrected tables here.

**Table 5:** Immediate outcome indicators for Preoperative Care in the Control Group 2009 to 2011.

Control group Preoperative Care	2009 t_1_ [%]	2010 t_2_ [%]	2011 t_3_ [%]	p value t_1_—t_2_	p value t_2_ –t_3_	p value t_1_ –t_3_
Was the patient seen by an anesthetist the day before operation?	7/32 [21.9]	0/24 [0]	0/24 [0]	0.012*↓	1	0.012*↓
Did she/he document the anesthetic history?	22/32 [68.8]	0/24 [0]	0/24 [0]	<0.001*↓	1	<0.001*↓
Have contributing illnesses been documented?	16/32 [50]	0/24 [0]	20/24 [83.3]	<0.001*↓	<0.001*↑	0.012*↑
Are allergies documented?	9/32 [28.1]	0/24 [0]	0/24 [0]	0.002*↓	1	0.002*↓
Are the necessary lab results documented (at least HB and FBS)?	25/32 [78.1]	8/24 [33.3]	16/24 [66.7]	<0.001*↓	0.042*↑	0.375
Did the patient get any form of premedication?	4/32 [12.5]	0/24 [0]	0/24 [0]	0.116	1	0.116
Was fasting ordered?	22/32 [68.8]	3/24 [12.5]	13/24 [54.2]	<0.001*↓	0.005*↑	0.282
Did the patient sign a consent form?	25/32 [78.1]	14/24 [58.3]	14/24 [58.3]	0.146	1	0.146
overall score [%]	**130/256 [50.8]**	**25/192 [13.02]**	**63/192 [32.8]**	<0.001*↓	<0.001*↑	<0.001*↓
difference of scores				0.3776	-0.1979	0.1797
95% CI of differences				0.2956–0.4505	-0.2781 – -0.1147	0.0875–0.2668

Immediate outcome indicators for Preoperative Care in the control group 2009–2011. Significance was tested 2010 (t2) against 2009 (t1), 2011 (t3) against 2010 (t2), and 2011(t3) against 2009 (t1). Significant improvement was marked with *↑, significant decline is marked with *↓ Confidence interval was given for the differences of overall immediate outcome of the key procedure in 2009, 2010, 2011. Resulting from the correction of Table 5, the following significant changes in immediate outcome indicators are new: significant improvement between t1 and t3 in “Have contributing illnesses been documented?”; significant improvement between t2 and t3 in “Are the necessary lab results documented (at least HB and FBS)?” HB = hemoglobin FBS = full blood survey; significant improvement between t2 and t3 in “Was fasting ordered”. The following result is not significant anymore after our correction: no significant change between t1 and t2 as well as between t1 and t3 in “Did the patient sign a consent form?”

**Table 7:** Immediate outcome indicators for Postoperative Inpatient Care in the Control Group 2009 to 2011.

Control group Postoperative Inpatient Care	2009 t_1_ [%]	2010 t_2_ [%]	2011 t_3_ [%]	p value t_1_—t_2_	p value t_2_ –t_3_	p value t_1_ –t_3_
Are orders fulfilled?	3/4 [75]	2/4 [50.0]	2/4 [50.0]	1	1	1
Are wounds dressed once a day?	2/4 [50]	2/4 [50.0]	2/4 [50.0]	1	1	1
Does staff wash or disinfect their hands after contact?	3/4 [75]	0/4 [0]	0/4 [0]	0.143	1	1
Does staff adhere to hygienic procedures when in contact with patients (when taking blood, putting up a drip etc.)?	3/4 [75]	2/4 [50.0]	2/4 [50.0]	1	1	1
Does staff document care?	3/4 [75]	2/4 [50.0]	2/4 [50.0]	1	1	1
Is the input /output checked every 4 hours for the first 24 hours (in case of major operation)?	17/34 [50]	6/18 [33.3]	19/26 [73.1]	0.379	0.014*↑	0.11
Is absence of bleeding checked at least twice?	15/34 [44.1]	3/18 [16.7]	7/26 [26.9]	0.068	0.489	0.036*↓
Is anti-pain medication applied?	34/34 [100]	17/18 [94.4]	26/26 [100]	0.346	0.409	n.a.
Does the patient have an (open) iv line?	27/34 [79.4]	18/18 [100]	26/26 [100]	0.081	1	0.081
Are notes and observation charts checked by Dr and documented once a day?	16/34 [47.1]	3/18 [16.7]	13/26 [50]	0.038*↓	<0.001*↓	<0.001*↓
**overall immediate outcome [%]**	**123/176 [69.9]**	**55/110 [50]**	**99/150 [66]**	0.001*↓	0.011	0.476
**difference**				0.1989	-0.1600	0.0389
**95% CI of differences**				0.0825–0.3102	-0.2961 – -0.0389	-0.0618–0.1398

Immediate outcome indicators for Preoperative Care in the control group 2009–2011. Significance was tested 2010 (t2) against 2009 (t1), 2011 (t3) against 2010 (t2), and 2011(t3) against 2009 (t1). Significant improvement was marked with *↑, significant decline is marked with *↓ Confidence interval was given for the differences of overall immediate outcome of the key procedure in 2009, 2010, 2011. Resulting from the correction of Table 7, the following significant changes in immediate outcome indicators are new: significant decline between t1 and t2 in “Are notes and observation charts checked by Dr and documented once a day?”.

The following information is missing from the Funding section: GIZ Deutsche Gesellschaft für Internationale Zusammenarbeit GmbH provided support in the form of grants to Dr. Mtatifikolo.

The following information is missing from the Competing Interests section: Dr. Mtatifikolo received grants from GIZ Deutsche Gesellschaft für Internationale Zusammenarbeit GmbH, a commercial company, during the conduct of this study.
