# Supplementary material for: Perioperative Care and the Importance of Continuous Quality Improvement—A Controlled Intervention Study in Three Tanzanian Hospitals
Source: PLoS One. 2015 Sep 1;10(9):e0136156. doi: 10.1371/journal.pone.0136156 (PMC4556680; doi:10.1371/journal.pone.0136156)
Supplement: S1 Table — (DOCX) [file pone.0136156.s001.docx]

| **DISTRICT:HANDENI** |  | **YEAR: 2009** |
| --- | --- | --- |

**Table 2: Demographic indicators** *(from computer printout: "Additional Reports MTUHA") (from table D 1.6)*

| Total population |  | **286.319** |  |
| --- | --- | --- | --- |
| Growth rate | 4,00% |  |  |
| Births (4.6%) | 4,60% | 11.445 |  |
| Children <1year (4.0%) | 4,00% | 10.589 |  |
| Children <5 years (20%) | 20,00% | 50.409 |  |
| Women 15-49 years (20%) | 20,00% | 68.906 |  |
|  | | | |

# 2.1 Health facilities, infrastructure, equipment

**Table 3: Health facilities per type and ownership and number of beds**

*(from computer printout: "Additional Reports MTUHA") (from F005 Part 1 and 4)*

Page 1

| Type of facility | **Government HF** | Nr of beds | **NGO HF** | Nr of beds | **Private HF** | Nr of beds | **Total Nr. HF** | **Total Nr. of beds** |
| --- | --- | --- | --- | --- | --- | --- | --- | --- |
| Hospitals | 1 | 119 | 1 | 56 | 0 | 0 | **2** | **175** |
| Health centers | 3 | 60 | 0 | 0 | 0 | 0 | **3** | **60** |
| Dispensaries | 31 |  | 1 |  | 3 |  | **35** | **0** |
| **TOTAL** | 35 | **179** | **2** | 56 | **3** | **0** | **40** | **235** |
| Comments:There is an increase of 1 dispensary from 30 to 31 health facility year 2009. | | | |  |  |  |  | |

**Table 4: Availability of amenities in health facilities** *(from table D 2.4)*

| Availability of amenities | Water | Electricity | Toilet | Refuse pit/placenta pit | Sewerage |  |
| --- | --- | --- | --- | --- | --- | --- |
| Nr of HF | 35 | 14 | 40 | 40 | 1 |  |
| *% of HF* | 87,50 | 35,00 | 100,00 | 100,00 | 2,50 |  |
| Comments:  All facilities have toilets and refuse pits. |  |  |  |  |  | |

**Table 6: Availability of essential equipment in working order** *(from table D 2.1)*

| Equipment | Adult scale | Baby scale | BP machine | Delivery kit | Fetoscope | Fridge | Sterilizer | Stethoscop e |
| --- | --- | --- | --- | --- | --- | --- | --- | --- |
| Nr of HF with at least one | 40 | 40 | 15 | 20 | 40 | 34 | 28 | 40 |

Page 2

| *% of HF with at least one* | 100,00 | 100,00 | 37,50 | 50,00 | 100,00 | 85,00 | 70,00 | 100,00 |
| --- | --- | --- | --- | --- | --- | --- | --- | --- |
| Comments:  Most health facilities have non functioning BP machines | |  |  |  |  |  |  |  |

# 2.2 Human resources

**Table 7 (a): District staff report – Only Government owned Institutions!**

*(from MTUHA Report Navigator: Reports – Resource Management – Annual Data – Staffing Data (from D001)*

| **Category** | **Requirement**    (according to "Staffing Levels for Health | | | |  | **Staff Available** | |  |
| --- | --- | --- | --- | --- | --- | --- | --- | --- |
|  | Gov.  Hospital /  CHMT | Gov. Rural  Health  Center | Gov. Dispensaries | **Total** | Gov.  Hospital /  CHMT | Gov. Rural  Health  Center | Gov. Dispensaries | **Total** |
| District Medical Officer | 1 | 0 | 0 | **1** | 0 | 0 | 0 | **0** |
| District Dental Officer | 1 | 0 | 0 | **1** | 1 | 0 | 0 | **1** |
| District Health Officer | 1 | 0 | 0 | **1** | 1 | 0 | 0 | **1** |
| District Nursing Officer | 1 | 0 | 0 | **1** | 1 | 0 | 0 | **1** |
| District Pharmacist | 1 | 0 | 0 | **1** | 1 | 0 | 0 | **1** |
| District Laboratory Technologist | 1 | 0 | 0 | **1** | 1 | 0 | 0 | **1** |
| District Health Secretary | 1 | 0 | 0 | **1** | 1 | 0 | 0 | **1** |
| Medical Doctor (incl. MO i/c) | 2 | 0 | 0 | **2** | 0 | 0 | 0 | **0** |
| Specialist Doctor | 0 | 0 | 0 | **0** | 0 | 0 | 0 | **0** |
| Dental Surgeon | 0 | 0 | 0 | **0** | 0 | 0 | 0 | **0** |
| Specialist Dental Surgeon | 0 | 0 | 0 | **0** | 0 | 0 | 0 | **0** |
| Pharmacist | 0 | 0 | 0 | **0** | 0 | 0 | 0 | **0** |
| Chemists | 0 | 0 | 0 | **0** |  |  |  | **0** |
| Assistant Medical Officer | 14 | 4 | 0 | **18** | 7 | 1 | 0 | **8** |
| Assistant Dental Officer | 1 | 0 | 0 | **1** | 1 | 0 | 0 | **1** |
| Medical Assistant / Clinical Officer | 32 | 12 | 74 | **118** | 16 | 6 | 22 | **44** |
| Dental assistant / Dental therapist | 1 | 0 | 0 | **1** | 0 | 0 | 0 | **0** |
| Rural Medical Aid | 0 | 0 | 0 | **0** | 0 | 0 | 6 | **6** |
| Nursing Officer / Public Health Nurse A | 36 | 6 | 0 | **42** | 13 | 2 | 1 | **16** |
| Nurse tutor | 0 | 0 | 0 | **0** | 0 | 0 | 0 | **0** |
| Trained Nurse/ Midwife/ Public Health Nurse B | 45 | 12 | 74 | **131** | 32 | 12 | 12 | **56** |
| MCH Aid | 0 | 8 | 0 | **8** | 1 | 2 | 5 | **8** |

Page 3

| Medical Laboratory Technician | 3 | 0 | 0 | **3** | 0 | 0 | 0 | **0** |
| --- | --- | --- | --- | --- | --- | --- | --- | --- |
| Radiographer | 1 | 0 | 0 | **1** | 1 | 0 | 0 | **1** |
| Dental Technician | 1 | 0 | 0 | **1** | 0 | 0 | 0 | **0** |
| Optometry Technician | 0 | 0 | 0 | **0** | 0 | 0 | 0 | **0** |
| Orthopedic Technician | 0 | 0 | 0 | **0** | 0 | 0 | 0 | **0** |
| Physiotherapist | 1 | 0 | 0 | **1** | 0 | 0 | 0 | **0** |
| Chemical Laboratory Technician | 0 | 0 | 0 | **0** | 0 | 0 | 0 | **0** |
| Health Officer |  |  |  | **0** |  | 0 | 8 | **8** |
| Medical Records Officers | 5 | 0 | 0 | **5** | 0 | 0 | 0 | **0** |
| Pharmaceutical Technician | 2 | 0 | 0 | **2** | 0 | 0 | 0 | **0** |
| Launderers | 4 | 0 | 0 | **4** | 0 | 0 | 0 | **0** |
| Catering officers | 0 | 0 | 0 | **0** | 0 | 0 | 0 | **0** |
| Health Secretary | 2 | 0 | 0 | **2** | 0 | 0 | 0 | **0** |
| Mortuary Attendant | 2 | 0 | 0 | **2** | 1 | 0 | 0 | **1** |
| Medical Attendant | 30 | 12 | 35 | **77** | 44 | 51 | 24 | **119** |
| All other | 23 | 27 | 0 | **50** | 8 | 0 | 0 | **8** |
| **TOTAL STAFF** | **212** | **81** | **183** | **476** | **130** | **74** | **78** | **282** |
| Comments:  Acute shortage of staff | | | | | |  |  |  |
| Comments: |  | | | |  | | | |

**4. In-Patient Data**

# 4.3 Special services

**Table 24: Surgical operations performed in District Hospital per type** (*from Theatre Register)*

| Major operations | Number | Minor operations | Number |  |
| --- | --- | --- | --- | --- |
| 1. Laparotomy | 93 | 1.  Evacuation | 143 |  |
| 2. Caesarian Section | 309 | 2. D&C | 18 |  |
| 3. Herniorrhaphy | 58 | 3.  Circumcision | 103 |  |
| 4. Hydrocelectomy | 45 | 4. Reduction of fracture | 354 |  |
| 5. Tubal ligation | 104 | 5. Surgical toilet | 136 |  |
| 6. Orchidectomy | 22 | 6. Other | 1120 |  |
| 7.Amputation | 9 |  |  |  |
| 8.Hysterectomy | 15 |  |  |  |
| 9. Ophthalmologic | 0 |  |  |  |
| 10. Other | 39 |  |  |  |
| **Total** | **694** | **Total** | **1874** |  |
| Comments:  Caesarian section has decreased from 339 in 2008 to 309 in 2009 | | | | |

Page 13
