# Supplementary material for: Perioperative Care and the Importance of Continuous Quality Improvement—A Controlled Intervention Study in Three Tanzanian Hospitals
Source: PLoS One. 2015 Sep 1;10(9):e0136156. doi: 10.1371/journal.pone.0136156 (PMC4556680; doi:10.1371/journal.pone.0136156)
Supplement: S2 Table — (DOCX) [file pone.0136156.s002.docx]

| **DISTRICT:HANDENI** |  | **YEAR: 2010** |
| --- | --- | --- |

**Table 2: Demographic indicators** *(from computer printout: "Additional Reports MTUHA") (from table D 1.6)*

Percentage

| Total population |  | **292.567** |  |
| --- | --- | --- | --- |
| Growth rate | 4,00% |  |  |
| Births | 4,60% | 11.541 |  |
| Children <1year | 4,00% | 10.695 |  |
| Children <5 years | 20,00% | 50.886 |  |
| Women 15-49 years | 20,00% | 70.852 |  |
| Comments:  These indicators are form NBS |  | | |

# 2.1 Health facilities, infrastructure, equipment

**Table 3: Health facilities per type and ownership and number of beds**

*(from computer printout: "Additional Reports MTUHA") (from F005 Part 1 and 4)*

Page 1

| Type of facility | **Government HF** | Nr of beds | **NGO HF** | Nr of beds | **Private HF** | Nr of beds | **Total Nr. HF** | **Total Nr. of beds** |
| --- | --- | --- | --- | --- | --- | --- | --- | --- |
| Hospitals | 1 | 120 | 1 | 56 | 0 | 0 | **2** | **176** |
| Health centers | 3 | 60 | 0 | 0 | 0 | 0 | **3** | **60** |
| Dispensaries | 33 | 0 | 1 | 0 | 3 | 0 | **37** | **0** |
| **TOTAL** | 37 | **180** | **2** | 56 | **3** | **0** | **42** | **236** |
| Comments:There is an increase of Health Facilities from 40 to42 health facilities year 2010. | | | |  |  |  |  | |

**Table 4: Availability of amenities in health facilities** *(from table D 2.4)*

| Availability of amenities | Water | Electricity | Toilet | Refuse pit/placenta pit | Sewerage |  |
| --- | --- | --- | --- | --- | --- | --- |
| Nr of HF | 42 | 14 | 42 | 42 | 1 |  |
| *% of HF* | 100,00 | 33,33 | 100,00 | 100,00 | 2,38 |  |
| Comments:  All facilities have toilets and refuse pits. |  |  |  |  |  | |

# 2.2 Human resources

**Table 7 (a): District staff report – Only Government owned Institutions!**

*(from MTUHA Report Navigator: Reports – Resource Management – Annual Data – Staffing Data (from D001)*

| **Category** | **Requirement** | | | |  | **Staff Available** | |  |
| --- | --- | --- | --- | --- | --- | --- | --- | --- |
|  | (according to "Staffing Levels for Health | | | |  |  | |  |
|  | Gov.  Hospital /  CHMT | Gov. Rural  Health  Center | Gov. Dispensaries | **Total** | Gov.  Hospital /  CHMT | Gov. Rural  Health  Center | Gov. Dispensaries | **Total** |
| District Medical Officer | 1 | 0 | 0 | **1** | 1 | 0 | 0 | **1** |
| District Dental Officer | 1 | 0 | 0 | **1** | 1 | 0 | 0 | **1** |
| District Health Officer | 1 | 0 | 0 | **1** | 1 | 0 | 0 | **1** |
| District Nursing Officer | 1 | 0 | 0 | **1** | 1 | 0 | 0 | **1** |
| District Pharmacist | 1 | 0 | 0 | **1** | 1 | 0 | 0 | **1** |
| District Laboratory Technologist | 1 | 0 | 0 | **1** | 1 | 0 | 0 | **1** |
| District Health Secretary | 1 | 0 | 0 | **1** | 1 | 0 | 0 | **1** |
| Medical Doctor (incl. MO i/c) | 2 | 0 | 0 | **2** | 0 | 0 | 0 | **0** |
| Specialist Doctor | 0 | 0 | 0 | **0** | 0 | 0 | 0 | **0** |
| Dental Surgeon | 0 | 0 | 0 | **0** | 0 | 0 | 0 | **0** |
| Specialist Dental Surgeon | 0 | 0 | 0 | **0** | 0 | 0 | 0 | **0** |
| Pharmacist | 0 | 0 | 0 | **0** | 0 | 0 | 0 | **0** |
| Chemists | 0 | 0 | 0 | **0** | 0 | 0 | 0 | **0** |
| Assistant Medical Officer | 14 | 4 | 0 | **18** | 10 | 2 | 0 | **12** |
| Assistant Dental Officer | 1 | 0 | 0 | **1** | 1 | 0 | 0 | **1** |
| Medical Assistant / Clinical Officer | 32 | 12 | 74 | **118** | 14 | 6 | 23 | **43** |
| Dental assistant / Dental therapist | 1 | 0 | 0 | **1** | 0 | 0 | 0 | **0** |
| Rural Medical Aid | 0 | 0 | 0 | **0** | 3 | 0 | 0 | **3** |
| Nursing Officer / Public Health Nurse A | 44 | 6 | 0 | **50** | 13 | 3 | 1 | **17** |
| Nurse tutor | 0 | 0 | 0 | **0** | 0 | 0 | 0 | **0** |
| Trained Nurse/ Midwife/ Public Health Nurse B | 100 | 12 | 74 | **186** | 35 | 10 | 13 | **58** |
| MCH Aid | 0 | 0 | 0 | **0** | 1 | 1 | 5 | **7** |

Page 3

| Medical Laboratory Technician | 3 | 0 | 0 | **3** | 1 | 0 | 0 | **1** |
| --- | --- | --- | --- | --- | --- | --- | --- | --- |
| Radiographer | 1 | 0 | 0 | **1** | 1 | 0 | 0 | **1** |
| Dental Technician | 1 | 0 | 0 | **1** | 0 | 0 | 0 | **0** |
| Optometry Technician | 0 | 0 | 0 | **0** | 0 | 0 | 0 | **0** |
| Orthopedic Technician | 0 | 0 | 0 | **0** | 0 | 0 | 0 | **0** |
| Physiotherapist | 1 | 0 | 0 | **1** | 0 | 0 | 0 | **0** |
| Chemical Laboratory Technician | 0 | 0 | 0 | **0** | 0 | 0 | 0 | **0** |
| Health Officer | 5 | 23 | 0 | **28** | 6 | 1 | 0 | **7** |
| Medical Records Officers | 5 | 0 | 0 | **5** | 0 | 0 | 0 | **0** |
| Pharmaceutical Technician | 2 | 0 | 0 | **2** | 0 | 0 | 0 | **0** |
| Launderers | 4 | 3 | 0 | **7** | 0 | 0 | 0 | **0** |
| Catering officers | 0 | 0 | 0 | **0** | 0 | 0 | 0 | **0** |
| Health Secretary | 1 | 0 | 0 | **1** | 0 | 0 | 0 | **0** |
| Mortuary Attendant | 2 | 0 | 0 | **2** | 1 | 0 | 0 | **1** |
| Medical Attendant | 72 | 12 | 70 | **154** | 30 | 21 | 37 | **88** |
| All other | 23 | 27 | 0 | **50** | 8 | 0 | 0 | **8** |
| **TOTAL STAFF** | **321** | **99** | **218** | **638** | **131** | **44** | **79** | **254** |
| Comments:  Acute shortage of trainned staff | | | | | |  |  |  |

**4. In-Patient Data**

# 4.3 Special services

**Table 24: Surgical operations performed in District Hospital per type** (*from Theatre Register)*

| Major operations | Number | Minor operations | Number |  |
| --- | --- | --- | --- | --- |
| 1. Laparotomy | 83 | 1.  Evacuation | 102 |  |
| 2. Caesarian Section | 410 | 2. D&C | 37 |  |
| 3. Herniorrhaphy | 51 | 3.  Circumcision | 115 |  |
| 4. Hydrocelectomy | 40 | 4. Reduction of fracture | 98 |  |
| 5. Tubal ligation | 113 | 5. Surgical toilet | 110 |  |
| 6. Orchidectomy | 4 | 6. Other | 2921 |  |
| 7.Amputation | 4 |  |  |  |
| 8.Hysterectomy | 16 |  |  |  |
| 9. Ophthalmologic | 0 |  |  |  |
| 10. Other | 254 |  |  |  |
| **Total** | **975** | **Total** | **3383** |  |
| Comments:  Caesarian section rate was in normal range - 7.8 % | | |  | |
