# Supplementary material for: Perioperative Care and the Importance of Continuous Quality Improvement—A Controlled Intervention Study in Three Tanzanian Hospitals
Source: PLoS One. 2015 Sep 1;10(9):e0136156. doi: 10.1371/journal.pone.0136156 (PMC4556680; doi:10.1371/journal.pone.0136156)
Supplement: S3 Table — (DOCX) [file pone.0136156.s003.docx]

| **DISTRICT:HANDENI** | **YEAR: 2011** |
| --- | --- |

**Table 2: Demographic indicators** *(from computer printout: "Additional Reports MTUHA") (from table D 1.6)*

Percentage

| Total population |  | **332.025** |  |
| --- | --- | --- | --- |
| Growth rate | 2,20% | **7.305** |  |
| Births (4.6%) | 4,60% | **15.273** |  |
| Children <1year (4.0%) | 4,00% | **13.281** |  |
| Children <5 years (21%) | 21,00% | **69.725** |  |
| Women 15-49 years (18%) | 18,00% | **59.765** |  |
| Comments: |  |  | |

# 2.1 Health facilities, infrastructure, equipment

**Table 3: Health facilities per type and ownership and number of beds**

*(from computer printout: "Additional Reports MTUHA") (from F005 Part 1 and 4)*

| Type of facility | **Govern-ment HF** | Nr of beds | **NGO HF** | Nr of beds | **Private HF** | Nr of beds |
| --- | --- | --- | --- | --- | --- | --- |
| Hospitals | 1 | 120 | 1 | 56 | 0 | 0 |
| Health centers | 3 | 60 | 0 | 0 | 0 | 0 |
| Dispensaries | 36 | 0 | 1 | 0 | 2 | 0 |
| **TOTAL** | 40 | **180** | **2** | 56 | **2** | **0** |
| Comments:  There is an increase of Health facilities from 42 to 44 health facilities this year 2011. | | |  |  |  |  |

**Table 4: Availability of amenities in health facilities** *(from table D 2.4)*

| Availability of amenities | Water | Electricity | Toilet | Refuse  pit/placenta pit | Sewerage |  |
| --- | --- | --- | --- | --- | --- | --- |
| Nr of HF | 44 | 23 | 44 | 44 | 2 |  |
| *% of HF* | 100 | 54,7 | 100 | 100 | 4% |  |
| Comments: 14 Health facilities being connected to electric supply from TANESCO while 9 are using solar power system | | | | | | |

**Table 6: Availability of essential equipment in working order** *(from table D 2.1)*

| Equipment | Adult scale | Baby scale | BP machine | Delivery kit | Fetoscope | Fridge |
| --- | --- | --- | --- | --- | --- | --- |
| Nr of HF with at least one | 25 | 42 | 23 | 30 | 44 | 34 |
| *% of HF with at least one* | 56,82 | 95,45 | 52,27 | 68,18 | 100,00 | 77,27 |
| Comments: | Most Health facilities have non functioning BP Machine | | |  |  |  |

# 2.2 Human resources

**Table 7 (a): District staff report – Only Government owned Institutions!**

*(from MTUHA Report Navigator: Reports – Resource Management – Annual Data – Staffing Data (from D001)*

| **Category** | **Requirement**    (according to "Staffing Levels for Health Facilities/Institutions" from MOH) | **Staff Av** |
| --- | --- | --- |

|  | Gov. Hospital /  CHMT | Gov. Rural Health Center | Gov. Dispen-saries | **Total** | Gov.  Hospital /  CHMT | Gov. Rural  Health  Center |
| --- | --- | --- | --- | --- | --- | --- |
| District Medical Officer | 1 | 0 |  | **1** | 1 | 0 |
| District Dental Officer | 1 | 0 | 0 | **1** | 1 | 0 |
| District Health Officer | 1 | 0 | 0 | **1** | 1 | 0 |
| District Nursing Officer | 1 | 0 | 0 | **1** | 1 | 0 |
| District Pharmacist | 1 | 0 | 0 | **1** | 1 | 0 |
| District Laboratory Technologist | 1 | 0 | 0 | **1** | 1 | 0 |
| District Health Secretary | 1 | 0 | 0 | **1** | 1 | 0 |
| Medical Doctor (incl. MO i/c) | 2 | 0 | 0 | **2** | 1 | 0 |
| Specialist Doctor | 0 | 0 | 0 | **0** | 0 | 0 |
| Dental Surgeon | 0 | 0 | 0 | **0** | 0 | 0 |
| Specialist Dental Surgeon | 0 | 0 | 0 | **0** | 0 | 0 |
| Pharmacist | 2 | 0 | 0 | **2** | 1 | 0 |
| Chemists | 0 | 0 | 0 | **0** | 0 | 0 |
| Assistant Medical Officer | 14 | 3 | 0 | **17** | 10 | 2 |
| Assistant Dental Officer | 1 | 0 | 0 | **1** | 1 | 0 |
| Medical Assistant / Clinical Officer | 32 | 12 | 74 | **118** | 12 | 6 |
| Dental assistant / Dental therapist | 1 | 0 | 0 | **1** | 0 | 0 |
| Rural Medical Aid | 0 | 0 | 0 | **0** | 0 | 0 |
| Nursing Officer / Public Health Nurse A | 44 | 6 | 0 | **50** | 14 | 3 |
| Nurse tutor | 0 | 0 | 0 | **0** | 0 | 0 |
| Trained Nurse/ Midwife/ Public Health Nurse B | 100 | 12 | 74 | **186** | 24 | 8 |
| MCH Aid | 0 | 0 | 0 | **0** | 1 | 1 |
| Medical Laboratory Technician | 3 | 0 | 0 | **3** | 2 | 0 |
| Radiographer | 1 | 0 | 0 | **1** | 1 | 0 |
| Dental Technician | 1 | 0 | 0 | **1** | 0 | 0 |
| Optometry Technician | 0 | 0 | 0 | **0** | 0 | 0 |
| Orthopedic Technician | 0 | 0 | 0 | **0** | 0 | 0 |
| Physiotherapist | 1 | 0 | 0 | **1** | 0 | 0 |
| Chemical Laboratory Technician | 0 | 0 | 0 | **0** | 0 | 0 |
| Health Officer | 5 | 23 | 0 | **28** | 5 | 5 |
| Medical Records Officers | 6 | 0 | 0 | **6** | 0 | 0 |
| Pharmaceutical Technician | 2 | 0 | 0 | **2** | 0 | 0 |
| Launderers | 4 | 3 | 0 | **7** | 0 | 0 |
| Catering officers | 0 | 0 | 0 | **0** | 0 | 0 |
| Health Secretary | 1 | 0 | 0 | **1** | 0 | 0 |
| Mortuary Attendant | 2 | 0 | 0 | **2** | 0 | 0 |
| Medical Attendant | 40 | 30 | 74 | **144** | 39 | 19 |
| All other |  |  |  | **0** | 2 | 0 |
| **TOTAL STAFF** | **269** | **89** | **222** | **580** | **120** | **44** |
| Comments:  Acute shortage of trained staff. |  |  |  |  |  |  |

**4. In-Patient Data**

# 4.3 Special services

**Table 24: Surgical operations performed in District Hospital per type** (*from Theatre Register)*

| Major operations | Number |  | Minor operations | Number |  |
| --- | --- | --- | --- | --- | --- |
| 1. Laparotomy |  | 66 | D&C | 0 |  |
| 2. Caesarian Section |  | 377 | Reduction/pop | 67 |  |
| 3. Herniorrhaphy |  | 67 | I&D |  |  |
| 4. Hydrocelectomy |  | 44 | Excision | 25 |  |
| 5. Tubal ligation |  | 489 | Deslouphing |  |  |
| 6. Orchidectomy |  | 4 | S/Toilet & suturing | 95 |  |
| 7.Amputation |  | 3 | FB removal |  |  |
| 8.Hysterectomy |  | 18 | S/pin insertion |  |  |
| 9. Ophthalmologic |  | 0 | Evacuation | 183 |  |
| 10. Other |  | 44 | Others | 77 |  |
| **Total** |  | **1112** | **Total** | **447** |  |
| Comments:  To tal major operations was1112 | |  | |  | |
