# Supplementary material for: Perioperative Care and the Importance of Continuous Quality Improvement—A Controlled Intervention Study in Three Tanzanian Hospitals
Source: PLoS One. 2015 Sep 1;10(9):e0136156. doi: 10.1371/journal.pone.0136156 (PMC4556680; doi:10.1371/journal.pone.0136156)
Supplement: S4 Table — (DOCX) [file pone.0136156.s004.docx]

| **DISTRICT: Lushoto** |  | **YEAR: 2009** |
| --- | --- | --- |

**Table 2: Demographic indicators** *(from computer printout: "Additional Reports MTUHA") (from table D 1.6)*

Percentage

| Total population |  | **487.132** |  |
| --- | --- | --- | --- |
| Growth rate | 1,10% |  |  |
| Births (4.6%) | 4,60% | 18.307 |  |
| Children <1year (4.0%) | 4,00% | 16.938 |  |
| Children <5 years (21%) | 21,00% | 78.849 |  |
| Women 15-49 years (18%) | 18,00% | 124.550 |  |
| Comments: Projected population. | | | |

# 2.1 Health facilities, infrastructure, equipment

**Table 3: Health facilities per type and ownership and number of beds**

*(from computer printout: "Additional Reports MTUHA") (from F005 Part 1 and 4)*

| Type of facility | **Government HF** | Nr of beds | **NGO HF** | Nr of beds | **Private HF** | Nr of beds | **Total Nr. HF** | **Total Nr. of beds** |  |
| --- | --- | --- | --- | --- | --- | --- | --- | --- | --- |
| Hospitals | 1 | 101 | 1 | 104 | 0 | 0 | **2** | **205** |  |
| Health centers | 6 | 94 | 1 | 37 | 0 | 0 | **7** | **131** |  |
| Dispensaries | 33 | 0 | 8 | 0 | 1 | 0 | **42** | **0** |  |
| **TOTAL** | 40 | **195** | **10** | 141 | **1** | **0** | **51** | **336** |  |
| Comments: Two NGO Dispensary closed due to manegerial problems. | | | | |  |  |  | | |

**Table 4: Availability of amenities in health facilities** *(from table D 2.4)*

| Availability of amenities | Water | Electricity | Toilet | Refuse pit/placenta pit | Sewerage |  |
| --- | --- | --- | --- | --- | --- | --- |
| Nr of HF | 50 | 21 | 50 | 50 | 7 |  |
| *% of HF* | 98,039216 | 41,18 | 98,03921569 | 98,039216 | 13,72549 |  |

**Table 6: Availability of essential equipment in working order** *(from table D 2.1)*

| Equipment | Adult scale | Baby scale | BP machine | Delivery kit | Fetoscope | Fridge | Sterilizer | Stethoscop e |  |
| --- | --- | --- | --- | --- | --- | --- | --- | --- | --- |
| Nr of HF with at least one | 47 | 48 | 51 | 25 | 48 | 46 | 51 | 51 |  |
| *% of HF with at least one* | 92,16 | 94,12 | 100,00 | 49,02 | 94,12 | 90,20 | 100,00 | 100,00 |  |
| Comments: | At least most of essential equipments are availlable at the health facilities. | | | | |  |  |  | |

# 2.2 Human resources

**Table 7 (a): District staff report – Only Government owned Institutions!**

*(from MTUHA Report Navigator: Reports – Resource Management – Annual Data – Staffing Data (from D001)*

| **Category** | **Requirement**    (according to "Staffing Levels for Health | | | |  | **Staff Available** | |  | **Over-**  **staffing /**  **Understaffing** |
| --- | --- | --- | --- | --- | --- | --- | --- | --- | --- |
|  | Gov.  Hospital /  CHMT | Gov. Rural Health Center | Gov. Dispensaries | **Total** | Gov.  Hospital /  CHMT | Gov. Rural  Health  Center | Gov. Dispensaries | **Total** | **Total** |
| District Medical Officer | 1 | 0 | 0 | **1** | 1 | 0 | 0 | **1** | **0** |
| District Dental Officer | 1 | 0 | 0 | **1** | 1 | 0 | 0 | **1** | **0** |
| District Health Officer | 1 | 0 | 0 | **1** | 1 | 0 | 0 | **1** | **0** |
| District Nursing Officer | 1 | 0 | 0 | **1** | 1 | 0 | 0 | **1** | **0** |
| District Pharmacist | 1 | 0 | 0 | **1** | 1 | 0 | 0 | **1** | **0** |
| District Laboratory Technologist | 1 | 0 | 0 | **1** | 1 | 0 | 0 | **1** | **0** |
| District Health Secretary | 1 | 0 | 0 | **1** | 1 | 0 | 0 | **1** | **0** |
| Medical Doctor (incl. MO i/c) | 1 | 0 | 0 | **1** | 0 | 0 | 0 | **0** | **-1** |
| Specialist Doctor | 0 | 0 | 0 | **0** | 0 | 0 | 0 | **0** | **0** |
| Dental Surgeon | 1 | 0 | 0 | **1** | 0 | 0 | 0 | **0** | **-1** |
| Specialist Dental Surgeon | 0 | 0 | 0 | **0** | 0 | 0 | 0 | **0** | **0** |
| Pharmacist | 1 | 0 | 0 | **1** | 1 | 0 | 0 | **1** | **0** |
| Chemists | 0 | 0 | 0 | **0** | 0 | 0 | 0 | **0** | **0** |
| Assistant Medical Officer | 5 | 7 | 0 | **12** | 9 | 0 | 0 | **9** | **-3** |
| Assistant Dental Officer | 1 | 0 | 0 | **1** | 0 | 0 | 0 | **0** | **-1** |
| Medical Assistant / Clinical Officer | 21 | 21 | 64 | **106** | 18 | 8 | 30 | **56** | **-50** |
| Dental assistant / Dental therapist | 1 | 7 | 0 | **8** | 1 | 0 | 0 | **1** | **-7** |
| Rural Medical Aid | 0 | 0 | 0 | **0** | 0 | 0 | 0 | **0** | **0** |
| Nursing Officer / Public Health Nurse A | 10 | 7 | 0 | **17** | 2 | 9 | 0 | **11** | **-6** |
| Nurse tutor | 0 | 0 | 0 | **0** | 0 | 0 | 0 | **0** | **0** |
| Trained Nurse/ Midwife/ Public Health Nurse B | 38 | 56 | 64 | **158** | 19 | 9 | 4 | **32** | **-126** |
| MCH Aid | 0 | 0 | 0 | **0** | 0 | 0 | 0 | **0** | **0** |
| Medical Laboratory Technician | 1 | 0 | 0 | **1** | 1 | 0 | 0 | **1** | **0** |
| Radiographer | 1 | 0 | 0 | **1** | 0 | 0 | 0 | **0** | **-1** |
| Dental Technician | 1 | 0 | 0 | **1** | 0 | 0 | 0 | **0** | **-1** |
| Optometry Technician | 1 | 0 | 0 | **1** | 0 | 0 | 0 | **0** | **-1** |
| Orthopedic Technician | 1 | 0 | 0 | **1** | 0 | 0 |  | **0** | **-1** |
| Physiotherapist | 1 | 0 | 0 | **1** | 0 | 0 | 0 | **0** | **-1** |
| Chemical Laboratory Technician | 0 | 0 | 0 | **0** | 0 | 0 | 0 | **0** | **0** |
| Health Officer | 6 | 7 | 0 | **13** | 1 | 5 | 0 | **6** | **-7** |
| Medical Records Officers | 2 | 7 | 0 | **9** | 2 | 0 | 0 | **2** | **-7** |
| Pharmaceutical Technician | 1 | 0 | 0 | **1** | 0 | 0 | 0 | **0** | **-1** |
| Launderers | 4 | 0 | 0 | **4** | 2 | 0 | 0 | **2** | **-2** |
| Catering officers | 0 | 0 | 0 | **0** | 0 | 0 | 0 | **0** | **0** |
| Health Secretary | 1 | 0 | 0 | **1** | 1 | 0 | 0 | **1** | **0** |
| Mortuary Attendant | 2 | 4 | 0 | **6** | 2 | 0 | 0 | **2** | **-4** |
| Medical Attendant | 25 | 28 | 32 | **85** | 40 | 59 | 64 | **163** | **78** |
| All other | 23 | 27 | 0 | **50** | 17 | 5 | 0 | **22** | **-28** |
| **TOTAL STAFF** | **156** | **171** | **160** | **487** | **123** | **95** | **98** | **316** | **-171** |
| Comments: Chronic shortage of staff at all level.(As per Establishment- IKAMA). | | | | | |  |  |  |  |

**4. In-Patient Data**

# 4.3 Special services

**Table 24: Surgical operations performed in District Hospital per type** (*from Theatre Register)*

| Major operations | Number | Minor operations | Number |  |
| --- | --- | --- | --- | --- |
| 1. Laparotomy | 38 | 1. Evacuation | 18 |  |
| 2. Caesarian Section | 287 | 2. D&C | 0 |  |
| 3. Herniorrhaphy | 25 | 3. Circumcision | 3 |  |
| 4. Hydrocelectomy | 11 | 4. Reduction of fracture | 290 |  |
| 5. Tubal ligation | 49 | 5. Surgical toilet | 135 |  |
| 6. Orchidectomy | 1 | 6. Other | 135 |  |
| 7.Amputation | 0 | M.V. A | 89 |  |
| 8.Hysterectomy | 6 | Foreign bodies | 3 |  |
| 9. Ophthalmologic | 162 |  |  |  |
| 10. Other | 38 |  |  |  |
| **Total** | **617** | **Total** | **673** |  |
| Comments: | There were slightly increase of caesarian section from263(2008) to 287. | | | |
