# Supplementary material for: Perioperative Care and the Importance of Continuous Quality Improvement—A Controlled Intervention Study in Three Tanzanian Hospitals
Source: PLoS One. 2015 Sep 1;10(9):e0136156. doi: 10.1371/journal.pone.0136156 (PMC4556680; doi:10.1371/journal.pone.0136156)
Supplement: S5 Table — (DOCX) [file pone.0136156.s005.docx]

| **DISTRICT:LUSHOTO** |  | **YEAR: 2010** |
| --- | --- | --- |

**Table 2: Demographic indicators** *(from computer printout: "Additional Reports MTUHA") (from table D 1.6)*

Percentage

| Total population |  | 496.917 |  |
| --- | --- | --- | --- |
| Growth rate | 4,00% |  |  |
| Births | 4,60% | 18.459 |  |
| Children <1year | 4,00% | 17.106 |  |
| Children <5 years | 20,00% | 79.589 |  |
| Women 15-49 years | 20,00% | 128.042 |  |
| Comments: | | | |

# 2.1 Health facilities, infrastructure, equipment

**Table 3: Health facilities per type and ownership and number of beds**

Page 1

*(from computer printout: "Additional Reports MTUHA") (from F005 Part 1 and 4)*

| Type of facility | **Government HF** | Nr of beds | **NGO HF** | Nr of beds | **Private HF** | Nr of beds | **Total Nr. HF** | **Total Nr. of beds** |
| --- | --- | --- | --- | --- | --- | --- | --- | --- |
| Hospitals | 1 | 109 | 1 | 120 | 0 | 0 | **2** | **229** |
| Health centers | 7 | 128 | 1 | 37 | 0 | 0 | **8** | **165** |
| Dispensaries | 32 | 0 | 9 | 0 | 1 | 0 | **42** | **0** |
| **TOTAL** | 40 | **237** | **11** | 157 | **1** | **0** | **52** | **394** |
| Comments:There is an increase of 1 dispensary from 31 to 32 health facility year 2010. | | | |  |  |  |  | |

**Table 4: Availability of amenities in health facilities** *(from table D 2.4)*

| Availability of amenities | Water | Electricity | Toilet | Refuse pit/placenta pit | Sewerage |  |
| --- | --- | --- | --- | --- | --- | --- |
| Nr of HF | 52 | 31 | 52 | 52 | 7 |  |
| *% of HF* | 100,00 | 59,62 | 100,00 | 100,00 | 13,46 |  |
| Comments:  10 health facilities instolled sollar electrification |  |  |  |  |  | |

**Table 6: Availability of essential equipment in working order** *(from table D 2.1)*

| Equipment | Adult scale | Baby scale | BP machine | Delivery kit | Fetoscope | Fridge | Sterilizer | Stethoscop e |
| --- | --- | --- | --- | --- | --- | --- | --- | --- |

Page 2

| Nr of HF with at least one | 52 | 48 | 52 | 48 | 48 | 46 | 52 | 52 |
| --- | --- | --- | --- | --- | --- | --- | --- | --- |
| *% of HF with at least one* | 100,00 | 92,31 | 100,00 | 92,31 | 92,31 | 88,46 | 100,00 | 100,00 |
| Comments:  There is increase of delivery kit from 25 to 48 and most of them was assembl | | | ed |  |  |  |  |  |

# 2.2 Human resources

**Table 7 (a): District staff report – Only Government owned Institutions!**

*(from MTUHA Report Navigator: Reports – Resource Management – Annual Data – Staffing Data (from D001)*

| **Category** | **Requirement**    (according to "Staffing Levels for Health | | | |  | **Staff Available** | |  |
| --- | --- | --- | --- | --- | --- | --- | --- | --- |
|  | Gov.  Hospital /  CHMT | Gov. Rural  Health  Center | Gov. Dispensaries | **Total** | Gov.  Hospital /  CHMT | Gov. Rural  Health  Center | Gov. Dispensaries | **Total** |
| District Medical Officer | 1 | 0 | 0 | **1** | 1 | 0 | 0 | **1** |
| District Dental Officer | 1 | 0 | 0 | **1** | 1 | 0 | 0 | **1** |
| District Health Officer | 1 | 0 | 0 | **1** | 1 | 0 | 0 | **1** |
| District Nursing Officer | 1 | 0 | 0 | **1** | 1 | 0 | 0 | **1** |
| District Pharmacist | 1 | 0 | 0 | **1** | 1 | 0 | 0 | **1** |
| District Laboratory Technologist | 1 | 0 | 0 | **1** | 1 | 0 | 0 | **1** |
| District Health Secretary | 1 | 0 | 0 | **1** | 1 | 0 | 0 | **1** |
| Medical Doctor (incl. MO i/c) | 1 | 0 | 0 | **1** | 1 | 0 | 0 | **1** |
| Specialist Doctor | 0 | 0 | 0 | **0** | 0 | 0 | 0 | **0** |
| Dental Surgeon | 1 | 0 | 0 | **1** | 0 | 0 | 0 | **0** |
| Specialist Dental Surgeon | 0 | 0 | 0 | **0** | 0 | 0 | 0 | **0** |
| Pharmacist | 1 | 0 | 0 | **1** | 0 | 0 | 0 | **0** |
| Chemists | 0 | 0 | 0 | **0** | 0 | 0 | 0 | **0** |
| Assistant Medical Officer | 5 | 7 | 0 | **12** | 13 | 0 | 0 | **13** |
| Assistant Dental Officer | 1 | 0 | 0 | **1** | 1 | 0 | 0 | **1** |
| Medical Assistant / Clinical Officer | 21 | 21 | 64 | **106** | 15 | 10 | 22 | **47** |
| Dental assistant / Dental therapist | 1 | 7 | 0 | **8** | 2 | 0 | 0 | **2** |
| Rural Medical Aid | 0 | 0 | 0 | **0** | 0 | 0 | 1 | **1** |
| Nursing Officer / Public Health Nurse A | 10 | 7 | 0 | **17** | 22 | 4 | 0 | **26** |
| Nurse tutor | 0 | 0 | 0 | **0** | 0 | 0 | 0 | **0** |
| Trained Nurse/ Midwife/ Public Health Nurse B | 38 | 56 | 64 | **158** | 24 | 6 | 7 | **37** |

Page 3

| MCH Aid | 0 | 0 | 0 | **0** | 2 | 4 | 12 | **18** |
| --- | --- | --- | --- | --- | --- | --- | --- | --- |
| Medical Laboratory Technician | 1 | 0 | 0 | **1** | 2 | 0 | 0 | **2** |
| Radiographer | 1 | 0 | 0 | **1** | 1 | 0 | 0 | **1** |
| Dental Technician | 1 | 0 | 0 | **1** | 0 | 0 | 0 | **0** |
| Optometry Technician | 1 | 0 | 0 | **1** | 0 | 0 | 0 | **0** |
| Orthopedic Technician | 1 | 0 | 0 | **1** | 0 | 0 | 0 | **0** |
| Physiotherapist | 1 | 0 | 0 | **1** | 0 | 0 | 0 | **0** |
| Chemical Laboratory Technician | 0 | 0 | 0 | **0** | 0 | 0 | 0 | **0** |
| Health Officer | 6 | 7 | 0 | **13** | 3 | 3 | 0 | **6** |
| Medical Records Officers | 2 | 7 | 0 | **9** | 0 | 0 | 0 | **0** |
| Pharmaceutical Technician | 1 | 0 | 0 | **1** | 0 | 0 | 0 | **0** |
| Launderers | 4 | 0 | 0 | **4** | 0 | 0 | 0 | **0** |
| Catering officers | 0 | 0 | 0 | **0** | 0 | 0 | 0 | **0** |
| Health Secretary | 1 | 0 | 0 | **1** | 1 | 0 | 0 | **1** |
| Mortuary Attendant | 2 | 4 | 0 | **6** | 2 | 0 | 0 | **2** |
| Medical Attendant | 25 | 28 | 32 | **85** | 48 | 39 | 58 | **145** |
| All other | 23 | 30 | 0 | **53** | 19 | 17 | 16 | **52** |
| **TOTAL STAFF** | **156** | **174** | **160** | **490** | **163** | **83** | **116** | **362** |
| Comments:  We have shortage of staff of about 26% of all cadres | | | | | |  |  |  |

**4. In-Patient Data**

Page 11

# 4.3 Special services

**Table 24: Surgical operations performed in District Hospital per type** (*from Theatre Register)*

| Major operations | Number | Minor operations | Number |  |
| --- | --- | --- | --- | --- |
| 1. Laparotomy | 36 | 1.  Evacuation | 15 |  |
| 2. Caesarian Section | 379 | 2. D&C | 21 |  |
| 3. Herniorrhaphy | 42 | 3.  Circumcision | 45 |  |
| 4. Hydrocelectomy | 13 | 4. Reduction of fracture | 48 |  |
| 5. Tubal ligation | 29 | 5. Surgical toilet | 57 |  |
| 6. Orchidectomy | 0 | 6. Other | 10 |  |
| 7.Amputation | 1 |  |  |  |
| 8.Hysterectomy | 5 |  |  |  |
| 9. Ophthalmologic | 94 |  |  |  |
| 10. Other |  |  | Page 13 |  |
| **Total** | **599** | **Total** | **196** |  |
| Comments:  There was an increase of caeserian section from 287 in 2009 to 397 in 2010. | | |  | |

Page 14
