# Supplementary material for: Perioperative Care and the Importance of Continuous Quality Improvement—A Controlled Intervention Study in Three Tanzanian Hospitals
Source: PLoS One. 2015 Sep 1;10(9):e0136156. doi: 10.1371/journal.pone.0136156 (PMC4556680; doi:10.1371/journal.pone.0136156)
Supplement: S6 Table — (DOCX) [file pone.0136156.s006.docx]

| **DISTRICT:LUSHOTO** | **YEAR: 2011** |
| --- | --- |

**Table 2: Demographic indicators** *(from computer printout: "Additional Reports MTUHA") (from table D 1.6)*

Percentage

| Total population |  | **507.303** |  |
| --- | --- | --- | --- |
| Growth rate | 2,20% | **11.161** |  |
| Births (4.6%) | 4,60% | **23.336** |  |
| Children <1year (4.0%) | 4,00% | **20.292** |  |
| Children <5 years (21%) | 21,00% | **106.534** |  |
| Women 15-49 years (18%) | 18,00% | **91.315** |  |
| Comments: |  |  | |

# 2.1 Health facilities, infrastructure, equipment

**Table 3: Health facilities per type and ownership and number of beds**

*(from computer printout: "Additional Reports MTUHA") (from F005 Part 1 and 4)*

| Type of facility | **Govern-ment HF** | Nr of beds | **NGO HF** | Nr of beds | **Private HF** | Nr of beds |
| --- | --- | --- | --- | --- | --- | --- |
| Hospitals | 1 | 109 | 1 | 120 | 0 | 0 |
| Health centers | 7 | 104 | 1 | 39 | 0 | 0 |
| Dispensaries | 34 | 0 | 9 | 0 | 1 | 0 |
| **TOTAL** | 42 | **213** | **11** | 159 | **1** | **0** |
| Comments: One dispensary was increased from 32 to 33 and | | | |  |  |  |

**Table 4: Availability of amenities in health facilities** *(from table D 2.4)*

| Availability of amenities | Water | Electricity | Toilet | Refuse  pit/placenta pit | Sewerage |  |
| --- | --- | --- | --- | --- | --- | --- |
| Nr of HF | 53 | 40 | 53 | 53 | 9 |  |
| *% of HF* | 100 | 76 | 100 | 100 | 17 |  |
| Comments: 31 Hfacilities being connected to electric supply from TANESCO while 9 they are using solar power system. | | | | | | |

**Table 6: Availability of essential equipment in working order** *(from table D 2.1)*

| Equipment | Adult scale | Baby scale | BP machine | Delivery kit | Fetoscope | Fridge |
| --- | --- | --- | --- | --- | --- | --- |
| Nr of HF with at least one | 50 | 43 | 53 | 35 | 53 | 48 |
| *% of HF with at least one* | 94,34 | 81,13 | 100,00 | 66,04 | 100,00 | 90,57 |
| Comments: | Only 35 (71%) out of 49 health facilies have delivery kits. | | |  |  |  |

# 2.2 Human resources

**Table 7 (a): District staff report – Only Government owned Institutions!**

*(from MTUHA Report Navigator: Reports – Resource Management – Annual Data – Staffing Data (from D001)*

| **Category** | **Requirement**    (according to "Staffing Levels for Health Facilities/Institutions" from MOH) | **Staff Av** |
| --- | --- | --- |

|  | Gov. Hospital /  CHMT | Gov. Rural Health Center | Gov. Dispen-saries | **Total** | Gov.  Hospital /  CHMT | Gov. Rural  Health  Center |
| --- | --- | --- | --- | --- | --- | --- |
| District Medical Officer | 1 | 0 | 0 | **1** | 1 | 0 |
| District Dental Officer | 1 | 0 | 0 | **1** | 1 | 0 |
| District Health Officer | 1 | 0 | 0 | **1** | 1 | 0 |
| District Nursing Officer | 1 | 0 | 0 | **1** | 1 | 0 |
| District Pharmacist | 1 | 0 | 0 | **1** | 1 | 0 |
| District Laboratory Technologist | 1 | 0 | 0 | **1** | 1 | 0 |
| District Health Secretary | 1 | 0 | 0 | **1** | 1 | 0 |
| Medical Doctor (incl. MO i/c) | 2 | 0 | 0 | **2** | 1 | 0 |
| Specialist Doctor | 0 | 0 | 0 | **0** | 0 | 0 |
| Dental Surgeon | 1 | 0 | 0 | **1** | 0 | 0 |
| Specialist Dental Surgeon | 0 | 0 | 0 | **0** | 0 | 0 |
| Pharmacist | 2 | 0 | 0 | **2** | 0 | 0 |
| Chemists | 0 | 0 | 0 | **0** | 0 | 0 |
| Assistant Medical Officer | 7 | 7 | 0 | **14** | 13 | 0 |
| Assistant Dental Officer | 1 | 0 | 0 | **1** | 1 | 0 |
| Medical Assistant / Clinical Officer | 21 | 21 | 64 | **106** | 18 | 8 |
| Dental assistant / Dental therapist | 1 | 7 | 0 | **8** | 2 | 0 |
| Rural Medical Aid | 0 | 0 | 0 | **0** | 0 | 0 |
| Nursing Officer / Public Health Nurse A | 10 | 7 | 0 | **17** | 19 | 3 |
| Nurse tutor | 0 | 0 | 0 | **0** | 0 | 0 |
| Trained Nurse/ Midwife/ Public Health Nurse B | 38 | 63 | 64 | **165** | 29 | 20 |
| MCH Aid | 0 | 0 | 0 | **0** | 0 | 0 |
| Medical Laboratory Technician | 1 | 0 | 0 | **1** | 2 | 0 |
| Radiographer | 1 | 0 | 0 | **1** | 1 | 0 |
| Dental Technician | 1 | 0 | 0 | **1** | 0 | 0 |
| Optometry Technician | 1 | 0 | 0 | **1** | 0 | 0 |
| Orthopedic Technician | 1 | 0 | 0 | **1** | 0 | 0 |
| Physiotherapist | 1 | 0 | 0 | **1** | 0 | 0 |
| Chemical Laboratory Technician | 0 | 0 | 0 | **0** | 0 | 0 |
| Health Officer | 6 | 7 | 0 | **13** | 3 | 3 |
| Medical Records Officers | 2 | 7 | 0 | **9** | 0 | 0 |
| Pharmaceutical Technician | 1 | 0 | 0 | **1** | 0 | 0 |
| Launderers | 4 | 0 | 0 | **4** | 0 | 0 |
| Catering officers | 0 | 0 | 0 | **0** | 0 | 0 |
| Health Secretary | 1 | 0 | 0 | **1** | 1 | 0 |
| Mortuary Attendant | 2 | 4 | 0 | **6** | 2 | 0 |
| Medical Attendant | 34 | 28 | 32 | **94** | 58 | 37 |
| All other | 23 | 30 | 0 | **53** | 9 | 7 |
| **TOTAL STAFF** | **169** | **181** | **160** | **510** | **166** | **78** |
| Comments: | | | | |  |  |

**4. In-Patient Data**

# 4.3 Special services

**Table 24: Surgical operations performed in District Hospital per type** (*from Theatre Register)*

| Major operations | Number | Minor operations | Number |  |
| --- | --- | --- | --- | --- |
| 1. Laparotomy | 25 | D&C | 20  129 |  |
| 2. Caesarian Section | 368 | Reduction/pop |  |  |
| 3. Herniorrhaphy | 34 | I&D | 43 |  |
| 4. Hydrocelectomy | 10 | Excision | 7 |  |
| 5. Tubal ligation | 26 | Dislouphing | 70 |  |
| 6. Orchidectomy | 0 | S/Toilet & suturing | 96 |  |
| 7.Amputation | 3 | FB removal | 6 |  |
| 8.Hysterectomy | 7 | S/pin insertion | 5 |  |
| 9. Ophthalmologic | 136 | Evacuation | 21 |  |
| 10. Other | 54 | Others | 610 |  |
| **Total** | **663** | **Total** | **987** |  |
| Comments:  There is slight reduction of caeserian section from 379 in (2010) to 368 (2011) | | |  | |
