# Supplementary material for: Perioperative Care and the Importance of Continuous Quality Improvement—A Controlled Intervention Study in Three Tanzanian Hospitals
Source: PLoS One. 2015 Sep 1;10(9):e0136156. doi: 10.1371/journal.pone.0136156 (PMC4556680; doi:10.1371/journal.pone.0136156)
Supplement: S7 Table — (PDF) [file pone.0136156.s007.pdf]

Supplement 1

# Intervention hospital – from the annual hospital reports

## 2009

**2.1. Human Resources:**

**Table 2. Hospital personnel - 2009**

| NO. | CADRE | Bombo Requirement | AVAILABLE | NATIONAL TARGET | NATIONAL TARGET VARIATION | | BOMBO REQUIREMENT VARIATION |
|---|---|---|---|---|---|---|---|
| | | | | | OVER | UNDER | |
| 1. | Medical Doctors | 10 | 9 | 7 | - | - | -1 |
| 2. | Specialist Doctors | 9 | 7 | 6 | - | - | -2 |
| 3. | Dental Surgeon | 2 | 3 | 2 | - | - | +1 |
| 4. | Pharmacist | 4 | 4 | 4 | - | - | - |
| 5. | Assistant Medical Officers | 18 | 18 | 14 | - | - | - |
| 6. | Assistant Dental Officers | 2 | 1 | 1 | - | - | -1 |
| 7. | Clinical Officers | 16 | 16 | 0 | - | - | - |
| 8. | Dental Auxiliary | 3 | 0 | 0 | - | - | -3 |
| 9. | Nursing Officers | 77 | 50 | 18 | - | - | -27 |
| 10. | Trained Nurses | 4 | 1 | 4 | - | 3 | -3 |
| 11. | Trained Midwives | 288 | 70 | 58 | - | 6 | -218 |
| 12. | Public Health Nurses | 10 | 4 | 6 | - | 2 | -12 |
| 13. | Medical Attendants | 145 | 133 | 51 | - | - | -12 |
| 14. | MCH Aid | 0 | 5 | 0 | - | +5 | +5 |
| 15. | Medical Laboratory Technician | 8 | 5 | 8 | - | 3 | -3 |
| 16. | Medical Laboratory Assistant | 0 | 0 | 0 | - | - | 0 |
| 17. | Radiographer | 3 | 3 | 3 | - | - | 0 |
| 18. | Optometry Technician | 2 | 2 | 2 | - | - | 0 |
| 19. | Health Officer | 4 | 5 | 4 | - | - | 0 |

| | CADRE | BOMBO REQUIREMENT | AVAILABLE | NATIONAL TARGET | NATIONAL TARGET VARIATION | | BOMBO REQUIREMENT VARIATION |
|---|---|---|---|---|---|---|---|
| | | | | | OVER | UNDER | |
| 20. | Pharmaceutical Technician | 7 | 2 | 2 | - | - | -5 |
| 21. | Pharmaceutical Assistant | 0 | 0 | 0 | 0 | - | |
| 22 | Purchasing Officer | 1 | 1 | 1 | - | - | - |
| 23. | Social Worker | 1 | 1 | 1 | - | - | - |
| 24 | Health Secretary | 1 | 1 | 1 | - | - | - |
| 25. | Mortuary Attendant | 4 | 1 | 4 | - | 3 | -3 |
| 26. | Technician (Medical Equipment) | 3 | 1 | 1 | - | - | -2 |
| 27. | Anaesthetic Officer | 3 | 1 | 2 | - | 1 | -2 |
| 28. | Launderer | 10 | 5 | 10 | - | 5 | -5 |
| 29. | Physiotherapist | 2 | 3 | 2 | - | - | - |
| 30. | Accountant | 1 | 1 | 1 | - | - | - |
| 32 | Accounts Assistant | 3 | 1 | 2 | - | -1 | -2 |
| 33. | Office Management Secretary/Typist | 2 | 1 | 2 | - | -1 | -1 |
| 34. | Registry Assistant and Record Management Assistant | 6 | 4 | 5 | - | -1 | -2 |
| 36. | Drivers | 6 | 7 | 6 | 1 | - | +1 |
| 37. | Storekeeper | 1 | 0 | 1 | - | 1 | -1 |
| 38. | Telephone Operator | 2 | 2 | 2 | - | - | - |
| 39 | Kitchen/Mess Attendant | 2 | 1 | 3 | - | 2 | -1 |
| 40.. | Office Attendant | 3 | 0 | 3 | - | 3 | -3 |
| 41 | Computer system analyst II | 1 | 1 | 1 | - | - | |
| **TOTAL** | | **664** | **370** | **238** | | - | |

| | **Bombo requirement** |
|---|---|
| **Standard** | 664 |
| **Available** | 370 |
| **Shortage** | 294 |

**Comments:** The hospital has now only 55% of the required workforce

### 3.2 Inpatient Summary

**Table 15: In-patient Utilization Summary**

| Department | Admissions | Deaths |
|---|---|---|
| Medical | 5316 | 297 |
| Surgical | 3035 | 172 |
| Gynaecology/Obstetrics | 7499 | 36 |
| Paediatrics - Neonatal Room | 4264 | 242 |
| **TOTAL** | **20114** | **747** |

### 5.4 Surgery Report- 2009

Summary:

- Total inpatient Male - 2901
- Total inpatient female - 714
- Total under fives. - 420
- Total Number of Deaths - 172

### 5.5 Anaesthetic Annual Report.

**OPERATIONS DONE FROM 1$^{ST}$ JANUARY – 31$^{ST}$ DECEMBER 2009**

**Total Number of Operations 2,432**

- Major Operations - 1495 = 61.5%
- Minor Operations - 937= 38.5%

| | | | | | |
|---|---|---|---|---|---|
| **2.** | Elective Operations | = | 1101 | = | 45.3% |
| **3.** | Emergency Operations | = | 1331 | = | 54.7% |

**Complications**

| | | | |
|---|---|---|---|
| 1. RESPIRATORY ARREST | - | 02 | |
| 2. MASSIVE BLOOD LOSS WITH HYPOTENSION AND ACUTE ANAEMIA | - | 03 | |
| 3. D.O.T | - | 05 | |
| 4. MASSIVE BLOOD LOSS WITH HYPOTENSION | - | 07 | |
| 5. HYPOTENSION | - | 11 | |
| 6. MASSIVE BLOOD LOSS | - | 18 | |
| TOTAL | - | 46 | **= 1.9%** |

# 2010

**2.2. Human Resources:**

**Table 2. Hospital personnel - 2010**

| No. | Cadre | Bombo Requirement | Available | National Target | National Target Variation | | Bombo Requirement Variation |
|---|---|---|---|---|---|---|---|
| | | | | | Over | Under | |
| 1. | Medical Doctors | 10 | 12 | 7 | - | - | +2 |
| 2. | Specialist Doctors | 9 | 5 | 6 | - | - | -4 |
| 3. | Dental Surgeon | 2 | 3 | 2 | - | - | +1 |
| 4. | Pharmacist | 4 | 3 | 4 | - | - | - 1 |
| 5. | Assistant Medical Officers | 18 | 21 | 14 | - | - | + 3 |
| 6. | Assistant Dental Officers | 2 | 1 | 1 | - | - | -1 |
| 7. | Clinical Officers | 16 | 17 | 0 | - | - | + 1 |
| 8. | Dental Auxiliary | 3 | 0 | 0 | - | - | -3 |
| 9. | Nursing Officers | 7 | 1 | 7 | - | - | -6 |
| 10. | Assistant Nursing Officers | 77 | 63 | 18 | - | - | -14 |
| 11. | Nurses | 288 | 73 | 58 | - | 6 | -215 |
| 12. | Public Health Nurses | 10 | 4 | 6 | - | 2 | -6 |
| 13. | Medical Attendants | 145 | 119 | 51 | - | - | -26 |
| 14. | Technologist (Lab, Xray, Eye &Pharm) | 21 | 16 | 8 | - | 3 | -5 |
| 15. | Assistant Technologist (Lab & Pharm) | 12 | 5 | 10 | - | - | -7 |
| 16. | Assistant Environmental Officer | 4 | 5 | 4 | - | - | +1 |

| No. | Cadre | Bombo Requirement | Available | National Target | National Target Variation | | Bombo Requirement Variation |
|---|---|---|---|---|---|---|---|
| | | | | | Over | Under | |
| 17. | Supplies Officer | 1 | 1 | 1 | - | - | - |
| 18. | Social Worker | 1 | 2 | 1 | - | - | + 1 |
| 19. | Health Secretary | 1 | 1 | 1 | - | - | - |
| 20. | Mortuary Attendant | 4 | 1 | 4 | - | 3 | -3 |
| 21. | Technician (Medical Equipment) | 3 | 2 | 1 | - | - | -1 |
| 22. | Anaesthetic Officer | 3 | 1 | 2 | - | 1 | -2 |
| 23. | Launderer | 10 | 4 | 10 | - | 5 | -6 |
| 24. | Physiotherapist | 2 | 3 | 2 | - | - | + 1 |
| 25. | Accountant | 1 | 1 | 1 | - | - | - |
| 26. | Accounts Assistant | 3 | 1 | 2 | - | -1 | -2 |
| 27. | Office Management Secretary/Typist | 2 | 1 | 2 | - | -1 | -1 |
| 28. | Registry Assistant and Record Management Assistant | 6 | 5 | 5 | - | -1 | -1 |
| 29. | Drivers | 6 | 7 | 6 | 1 | - | +1 |
| 30. | Storekeeper | 1 | 0 | 1 | - | 1 | -1 |
| 31. | Telephone Operator | 2 | 2 | 2 | - | - | - |
| 32 | Kitchen/Mess Attendant | 2 | 1 | 3 | - | 2 | -1 |
| 33. | Office Attendant | 3 | 0 | 3 | - | 3 | -3 |
| 34. | Computer system analyst II | 1 | 1 | 1 | - | - | |
| **TOTAL** | | **679** | **382** | **307** | | - | |

| | **Bombo requirement** |
|---|---|
| **Standard** | 680 |
| **Available** | 382 |
| **Shortage** | 298 |

**Comments:** The hospital has now only 56 % of the required human resource.

**SURGERY ANNUAL REPORT 2010**

**Summary**

- Total Number of patients 3654

  Males In- patients 2559

  Females In- patients 1095

- Total under five childrens 475

  Males 276

  Females 203

- Total Number of Deaths 107

## CHALLENGES

- Shortage of working equipment/ tools e.g. bed sheets, dressing trays, movable trolleys and beds.
- Lack of permanent departmental specialists / surgeons.
- Shortage of staffs i.e. Trained nurses

**SUGGESTIONS**

- Hospital to employ permanent surgeon
- Hospital to improve availability of working equipment and trained staffs

## 5.7. Anaesthetic Annual Report.

**OPERATIONS DONE FROM 1ST JANUARY – 31ST DECEMBER 2010**

**Total Number of Operations 2,375**

- Major Operations - 1,558 = 6562%
- Minor Operations - 817 = 34.4%

4. Elective Operations = 1044 = 44%
5. Emergency Operations = 1331 = 56%

**COMPLICATIONS**

1. Massive Blood loss - 07
2. Hypotension - 01
3. Massive Blood loss with hypotension - 02
4. Massive Blood loss/Acute Anaemia/hypotension - 10
5. Cardial Arrest - 02
6. Post Oper Death - 02
7. D.O.T - 02

**TOTAL 26 =1.1%**

# 2011

## 2.3. Human Resources:

**Table 2. Hospital personnel - 2011**

| No. | Cadre | Bombo Requirement | Available | National Target | National Target Variation | | Bombo Requirement Variation |
|---|---|---|---|---|---|---|---|
| | | | | | Over | Under | |
| 1. | Medical Doctors | 10 | 12 | 7 | - | - | +2 |
| 2. | Specialist Doctors | 9 | 5 | 6 | - | - | -4 |
| 3. | Dental Surgeon | 2 | 3 | 2 | - | - | +1 |
| 4. | Pharmacist | 4 | 3 | 4 | - | - | - 1 |
| 5. | Assistant Medical Officers | 18 | 21 | 14 | - | - | + 3 |
| 6. | Assistant Dental Officers | 2 | 1 | 1 | - | - | -1 |
| 7. | Clinical Officers | 16 | 17 | 0 | - | - | + 1 |
| 8. | Dental Auxiliary | 3 | 0 | 0 | - | - | -3 |
| 9. | Nursing Officers | 7 | 1 | 7 | - | - | -6 |
| 10. | Assistant Nursing Officers | 77 | 63 | 18 | - | - | -14 |
| 11. | Nurses | 288 | 73 | 58 | - | 6 | -215 |
| 12. | Public Health Nurses | 10 | 4 | 6 | - | 2 | -6 |
| 13. | Medical Attendants | 145 | 119 | 51 | - | - | -26 |
| 14. | Technologist (Lab, Xray, Eye &Pharm) | 21 | 16 | 8 | - | 3 | -5 |
| 15. | Assistant Technologist (Lab & Pharm) | 12 | 5 | 10 | - | - | -7 |
| 16. | Assistant Environmental Officer | 4 | 5 | 4 | - | - | +1 |

| No. | Cadre | Bombo Requirement | Available | National Target | National Target Variation | | Bombo Requirement Variation |
|---|---|---|---|---|---|---|---|
| | | | | | Over | Under | |
| 17. | Supplies Officer | 1 | 1 | 1 | - | - | - |
| 18. | Social Worker | 1 | 2 | 1 | - | - | + 1 |
| 19. | Health Secretary | 1 | 1 | 1 | - | - | - |
| 20. | Mortuary Attendant | 4 | 1 | 4 | - | 3 | -3 |
| 21. | Technician (Medical Equipment) | 3 | 2 | 1 | - | - | -1 |
| 22. | Anaesthetic Officer | 3 | 1 | 2 | - | 1 | -2 |
| 23. | Launderer | 10 | 4 | 10 | - | 5 | -6 |
| 24. | Physiotherapist | 2 | 3 | 2 | - | - | + 1 |
| 25. | Accountant | 1 | 1 | 1 | - | - | - |
| 26. | Accounts Assistant | 3 | 1 | 2 | - | -1 | -2 |
| 27. | Office Management Secretary/Typist | 2 | 1 | 2 | - | -1 | -1 |
| 28. | Registry Assistant and Record Management Assistant | 6 | 5 | 5 | - | -1 | -1 |
| 29. | Drivers | 6 | 7 | 6 | 1 | - | +1 |
| 30. | Storekeeper | 1 | 0 | 1 | - | 1 | -1 |
| 31. | Telephone Operator | 2 | 2 | 2 | - | - | - |
| 32 | Kitchen/Mess Attendant | 2 | 1 | 3 | - | 2 | -1 |
| 33. | Office Attendant | 3 | 0 | 3 | - | 3 | -3 |
| 34. | Computer system analyst II | 1 | 1 | 1 | - | - | |
| **TOTAL** | | **679** | **382** | **307** | | - | |

**SURGERY ANNUAL REPORT 2011**

**Summary**

| | |
|---|---|
| • Total Number of patients | 3654 |
| Males In- patients | 2559 |
| Females In- patients | 1095 |
| • Total under five childrens | 475 |
| Males | 276 |
| Females | 203 |
| • Total Number of Deaths | 107 |

**OPERATIONS DONE FROM 1ST JANUARY – 31ST DECEMBER 2011**

**Total Number of Operations 2,375**

- Major Operations - 1,558 = 6562%
- Minor Operations - 817 = 34.4%

| | | | | | |
|---|---|---|---|---|---|
| **6.** | Elective Operations | = | 1044 | = | 44% |
| **7.** | Emergency Operations | = | 1331 | = | 56% |

**COMPLICATIONS**

**8.** Massive Blood loss - 07
**9.** Hypotension - 01
**10.** Massive Blood loss with hypotension - 02
**11.** Massive Blood loss/Acute Anaemia/hypotension - 10
**12.** Cardial Arrest - 02
**13.** Post Oper Death - 02
**14.** D.O.T - 02

**TOTAL 26 =1.1%**
